# Advanced imaging-enabled understanding of cell wall remodeling mechanisms mediating plant drought stress tolerance

**DOI:** 10.3389/fpls.2025.1635078

**Published:** 2025-08-08

**Authors:** Nannan Zhao, Zhiguo Zhou, Shunli Cui, Xinye Zhang, Shu Zhu, Ying Wang, Tinashe Zenda, Li Wenjing

**Affiliations:** ^1^ College of Life Science, Langfang Normal University, Langfang, Hebei, China; ^2^ State Key Laboratory of North China Crop Improvement and Regulation, Hebei Agricultural University, Baoding, China; ^3^ Deparment of Crop Science, Faculty of Plant and Animal Sciences and Technology, Marondera University of Agricultural Sciences and Technology, Marondera, Zimbabwe

**Keywords:** cell wall modifications, drought tolerance, pectin methyltransferases, stomata guard cell wall, lignification, cell wall imaging

## Abstract

Drought stress causes peculiar challenges to plant cells reliant on turgor pressure and a polysaccharides-enriched cell wall for growth and development. Appropriate cell wall changes in mechanical properties and biochemical composition under stress conditions constitute an indispensable stress adaptation strategy. A better understanding of stress-induced cell wall modifications is not only crucial for accruing fundamental scientific knowledge in plant biology, but will help us design novel strategies for enhancing crop drought tolerance. Here, we extensively reviewed how selected cell wall remodeling mechanisms, including cell wall demethylesterification, cell wall loosening and stiffening, stomata guard cell wall adjustment, cell wall lignification and root cell wall suberization orchestrate plant drought tolerance, revealing a potential target area for drought tolerance improvement in crops. Stress-induced demethylesterification of pectins, mediated by pectin methylesterases, permits calcium crosslinking of polyphenolics, which enhances cell wall rigidity and may help in intra-cell water preservation. Cell wall proteins such as xyloglucan endotransglucosylases/hydrolase, β-glucanases and expansins are regulated by drought stress, and orchestrate cell turgor-driven cell expansion, through modulating the loosening of cell wall polysaccharides, enabling cell and organ growth under those conditions. Meanwhile, overexpression of certain cell wall proteins/genes such as expansins may promote drought tolerance by improving cell water retention, antioxidant capacity, water use efficiency, and osmotic adjustment. We also discuss the genetic, transcriptional, and phytohormonal regulations of cell wall remodeling. Further, we highlight the recent advancements in elucidation of plant cell wall biosynthesis as aided by cutting-edge high-resolution imaging techniques that now facilitate direct visualization and quantitative *in-situ* (real-time) microanalysis of cell wall chemical composition and dynamics. Integrating latest cell wall imaging techniques to innovative single-cell omics, genome editing, and advanced data analysis approaches could facilitate appropriate cell wall modifications necessary for drought tolerance engineering in crop plants.

## 1 Introduction

Plants constantly encounter harsh environmental conditions throughout their lifespan. Among these conditions, drought stress is the primary abiotic factor hindering plant growth, development, and productivity, threatening crop production (Martignago et al., 2020; Bashir et al., 2021). Drought is often defined in a meteorological sense to mean a period of below normal precipitation that restricts plant growth and productivity in a natural or agricultural system (Boyer, 1982). However, drought stress (more relevant to plant physiology and referred to herein) is a different concept which refers to a plant water status when there is reduced water available for the plant, due to a decrease in water potential (ψw, or the free energy of water) and turgor, that is enough to disrupt the normal plant physiological functioning (Kramer and Boyer, 1995; Osmolovskaya et al., 2018; Fradera-Soler et al., 2022). Therefore, drought stress responses often mean responses to an altered plant water status due to reduced plant available water (as water acquires a lower free energy state in relation to unstressed conditions) (Verslues et al., 2006; Juenger and Verslues, 2023). Similar to plant cell turgor, drought stress is often quantified in pressure terms (as a decrease in ψw) (Kramer and Boyer, 1995; Verslues et al., 2006), permitting an assessment of the two to be made to establish the plant-water relationship and the direction of water movement along the soil-plant-continuum (Juenger and Verslues, 2023). Meanwhile, decreased ψw complicates the ability of the plant to take up water, which in turn prompts a repertoire of responses that enable the plant to avoid water loss, permit continual water uptake at decreased ψw, or help the plant to tolerate a state of reduced tissue water content (Verslues et al., 2006; Osmolovskaya et al., 2018). The severity of drought stress on a crop depends on its intensity and duration, genotypic capacity of species to resist, the plant developmental stage at occurrence, and the plant tissue affected (Zia et al., 2021). Whilst “drought” naturally causes drought stress over time, a short period (of just few days or even one day) without water may be sufficient enough to cause water deficit (drought stress) that negatively impact yield (Shao et al., 2009; Cluzel, 2024; Vadez et al., 2024), especially if it occurs at the critical stage of plant development such as anthesis or grain filling stages in maize (Vennam et al., 2023). The physiological effects of drought stress on plants include decreased leaf water potential, loss of cell turgor, disrupted plant water relations, reactive oxygen species (ROS) over-accumulation, impaired photosynthesis, inhibited cell growth, impaired metabolism of cell wall components, compromised stomatal functioning, etc. (detailed in (Le Gall et al., 2015; Osmolovskaya et al., 2018; Gupta et al., 2020)). In concert, all these factors retard plant growth and development, and decrease crop yields. Therefore, the increased drought incidences and severity associated with climate change (Osmolovskaya et al., 2018; Means, 2023), often causing moderate to severe drought stress that limit crop productivity and threatens global food security (Vadez et al., 2024), motivate the need to develop drought stress-tolerant crops for sustainable food production. This depends on first gaining a mechanistic understanding of how plants respond and adapt to such stress (Zhang et al., 2022).

Plants respond to drought stress by evoking elaborate cellular, physiological, biochemical, and anatomical changes, including cell wall modifications, root architectural and biochemical adjustments, phytohormonal elicitation, etc (Gupta et al., 2020; Seleiman et al., 2021). These morpho-physiological responses are tightly regulated by genetic and molecular mechanisms (Bashir et al., 2021; Liu and Qin, 2021). In particular, the cell wall is a complex and dynamic entity whose properties are tightly regulated via cell wall remodeling (see **Table 1** for definitions), which refers to controlled modification, rearrangement, degradation or/and reconstruction of the cell wall in both growing and mature cells in response to various cues (Barnes and Anderson, 2018; Fradera-Soler et al., 2022). These modifications include cell wall loosening, cell wall stiffening, etc. and can be in response to biotic (Bacete et al., 2018; Swaminathan et al., 2022; Molina et al., 2024a) or abiotic stresses (Moore et al., 2008; Tenhaken, 2014; Oliveira et al., 2020; Jardine et al., 2022; Li et al., 2024) and/or developmental cues. For instance, cell wall components such as cellulose, hemicellulose and pectin are dynamically remodeled as an immune response against pathogen infection (Wan et al., 2021). Upon infection, cell wall hydrolysis induced by cell wall degrading enzymes releases carbohydrates (gylcans) that are sensed by plant receptors as alert signals to trigger plant immune response (Molina et al., 2024a; Molina et al., 2024b). Thus, infection-induced cell wall modification crucially mediates plant defense signaling and pathogen resistance (Wan et al., 2021; Molina et al., 2024a). Besides modulating biotic stress response, cell wall remodeling essentially mediates abiotic stress resistance in plants (Tenhaken, 2014; Houston et al., 2016; Jardine et al., 2022). In some instances, pathogen-triggered cell wall-related immune responses may partially overlap with abiotic (eg. salinity) stress-induced adaptive mechanisms, offering an exquisite machinery for combined stress resistance (Gigli-Bisceglia and Testerink, 2021), Thus, cell wall remodelng is an indispensable plant stress adaptation strategy.

**Table 1:** Definition of key terms related to cell wall remodeling. .

Term	Definition	References
Cell wall remodeling	Controlled modification, rearrangement, degradation or/and reconstruction of the cell wall in both growing and mature cells in response to various cues, including abiotic and biotic stress, or developmental cues.	(Barnes and Anderson, 2018; Fradera-Soler et al., 2022)
Cell wall stiffening	A process of making the cell wall rigid, orchestrated by laccases- and peroxidases-generated ROS (oxygen radicals, ·OH), and/or demethylesterification of pectic homogalacturonans (HGs) which culminates in Ca^2+^ crosslinking of HGs to form Ca^2+^–pectate cross-linked complexes.	(Vanholme et al., 2010; Peaucelle et al., 2011; Hoffmann et al., 2020)
Cell wall loosening	The process of relaxation of turgor-induced cell wall tension, aided by the actions of cell wall proteins such as XTHs, XETs, β-glucanases and expansins as they hydrolize cell wall polysaccharide substrates in a pH-dependent manner, permitting creep or/and turgor-driven cell enlargement.	(Cosgrove, 2000; Cosgrove, 2016a; Cosgrove, 2018; Zhang et al., 2019)
Creep	Irreversible time-dependent extension of the cell wall after it has been subjected to a certain level of strain.	(Cosgrove, 2016a; Cosgrove, 2018; Zhang et al., 2019)
Demethylesterification (of pectin)	Modification of pectin, catalysed by pectin methylesterases, which involves the removal of methyl esters from the d-GalA backbone of pectic HGs, often resulting in Ca^2+^ crosslinking of HGs to form a pectate gel matrix and increased cell wall stiffness.	(Peaucelle et al., 2011; Hongo et al., 2012; Wu et al., 2018)
Elasticity modulus (ϵ)	An index that measures the cell wall's stress/strain ratio, where strain is proportional to stress and is fully and immediately reversible upon reduction of stress. It measures the cell wall`s unique ability to expand without breaking or weakening.	(Cosgrove, 2016a; Cosgrove, 2016b)
Lignification	Involves the deposition of lignin, phenolic polymers, in apoplastic cell wall domains, rendering them mechanically strong, firm, and hydrophobic.	(Vanholme et al., 2010)

Essentially, drought stress results in loss of cell turgor, decreased leaf and root water potentials, osmotic adjustment, decreased stomatal conductance, etc (Osmolovskaya et al., 2018; Hemati et al., 2022). Since turgor pressure drives cell expansion and growth (dependent upon cell wall extensibility), drought stress-triggered reduction in cell turgor pressure leads to reduced or ceased growth, due to reduced cell wall extensibility and cell expansion (Le Gall et al., 2015). In response, plant cell walls undergo dynamic mechanical and chemical composition modifications to cope with these drought stress effects (Tenhaken, 2014; Le Gall et al., 2015). Besides, drought stress, just like other abiotic or biotic stresses, induces cell wall damages, such as cellulose damage or reduction, or pectin breakdown, etc., which compromise the cell wall integrity (CWI) and proper functioning of the cell. These changes prompt appropriate cell wall damage responses, including cell wall stress sensing and signaling, cell wall remodeling mechanisms, and activation of downstream gene expressions and physiological responses, to ensure CWI maintenance and plant adaptation to drought stress (Le Gall et al., 2015; Vaahtera et al., 2019). In some instances, the plant cell wall elasticity is critical in facilitating differential root cell wall responses, such as loosening and stiffening within different root zones, necessary for continued root growth under drought stress conditions (Wu and Cosgrove, 2000; Cosgrove, 2016a). Furthermore, the guard cell wall is dynamically remodeled, for instance, through differential thickening and orientation of cellulose microfibrils, to permit continual stomatal opening and closing necessary for adaptation to different drought stress episodes (Amsbury et al., 2016; Jaafar and Anderson, 2024).

Cell wall remodeling processes are regulated by several loosening and stiffening enzymes/proteins, including pectin methylesterases (PMEs) which catalyze cell-wall esters hydrolysis, α-expansins (EXPAs), β-glucanases, peroxidases (PODs), etc (Tenhaken, 2014; Chebli and Geitmann, 2017; Wu et al., 2018; Perrot et al., 2022), with the resultant physicochemical shifts being critical for proper cell and tissue morphogenesis and stress adaptation (Chebli and Geitmann, 2017; Jardine et al., 2022). The activities of these cell wall remodeling-involved enzymes are tightly controlled, spatio-temporally (Barnes and Anderson, 2018). Besides, different phytohormones such as auxins (Jobert et al., 2023) and brassinosteroids (BRs) (Rao and Dixon, 2017) precisely regulate and potentiate the transcriptional output, actions and modulation of genes encoding cell wall remodeling-involved enzymes (Jobert et al., 2023). Meanwhile, several papers have uncovered the role of cell wall remodeling in enhancing plant abiotic stress tolerance, including salinity (Gigli-Bisceglia and Testerink, 2021; Dabravolski and Isayenkov, 2023), cold (Bilska-Kos et al., 2022; Kutsuno et al., 2023), cadmium (Loix et al., 2017), and heat (Wu H-C. et al., 2017; Wu et al., 2018), among others (Le Gall et al., 2015; Ezquer et al., 2020; García-Angulo and Largo-Gosens, 2022). For example, negatively charged pectin effectively sequester cadmium whereas lignification immobilizes cadmium to enhance cadmium tolerance in plants (Loix et al., 2017). Cell wall integrity (CWI) maintenance, lignin accumulation and amplified ascorbate-mediated antioxidant defense help plants adapt to salinity (Rui and Dinneny, 2020; Liu J. et al., 2021; Dabravolski and Isayenkov, 2023). Increased expression of lignin biosynthesis-related enzymes (such as peroxidases), and other cell-wall related proteins (including expansins) enhances thermotolerance acquisition (Wu and Jinn, 2010; Tenhaken, 2014; Wu et al., 2018). Thus, apt stress-induced cell wall compositional shifts constitute an effective strategy regulating plant abiotic stress adaptation (Oliveira et al., 2020). Despite all these examples revealing the role of cell wall remodeling in plant abiotic stress resistance, relatively less focus has been placed on its role in drought tolerance. Thus, our knowledge on how stress-induced cell wall modifications mediate drought tolerance remains fragmented.

More recently, an increased number of researches have revealed the role of cell wall remodeling as an important strategy for drought stress response and adaptation in several plant species. For example, cell wall *O*-acetylation (through enhanced *O*-acetyl esters, but down-regulated methyl ester hydrolysis) under drought stress in poplar (*Populus trichocarpa*) fine-tunes cell wall elasticity, regulates proper vascular tissue functioning, and influences growth-stress response trade-offs (Jardine et al., 2022). In soybean (*Glycine max*), increased cell wall plasticity and crosslinking under drought contribute to improved hydraulic conductance, water use efficiency, photosynthesis performance, and sustained higher plant growth under stress (Coutinho et al., 2021). Meanwhile, environmental-acclimation-triggered leaf cell wall modifications in *Vitis vinifera* have been suggested to crucially regulate leaf physiology by markedly affecting photosynthesis and water relations in an environmental condition-dependent manner (Roig-Oliver et al., 2020). These examples buttress the significant role of cell wall remodeling as a plant drought response strategy; hence, a budding area for research, and a promising trait for enhancing crop drought tolerance and yield (Ganie and Ahammed, 2021; García-Angulo and Largo-Gosens, 2022). Therefore, a better understanding of the stress-induced plant cell wall modifications may help us create novel strategies for enhancing crop drought tolerance (Piccinini et al., 2024).

In this review, we parse together the recent insights on how selected cell wall remodeling mechanisms, including cell wall demethylesterification, cell wall loosening and stiffening, stomata guard cell adjustment, cell wall lignification and root cell wall suberization orchestrate plant drought tolerance, revealing a potential target area for drought tolerance improvement in crops. First, we describe the primary structural composition of cell wall, cell wall-related sensing and signaling systems governing abiotic stress response, and forms of stress-induced cell wall modifications. We also review phytohormonal regulation of cell wall modifications that orchestrate drought tolerance in plants. Further, we highlight the recent advances in cell wall imaging techniques that now facilitate direct visualization and real-time quantification of native cell wall chemical composition and dynamics. We sum up by proffering future prospects related to the topic.

## 2 Cell wall composition and architecture

**Figure 1:**
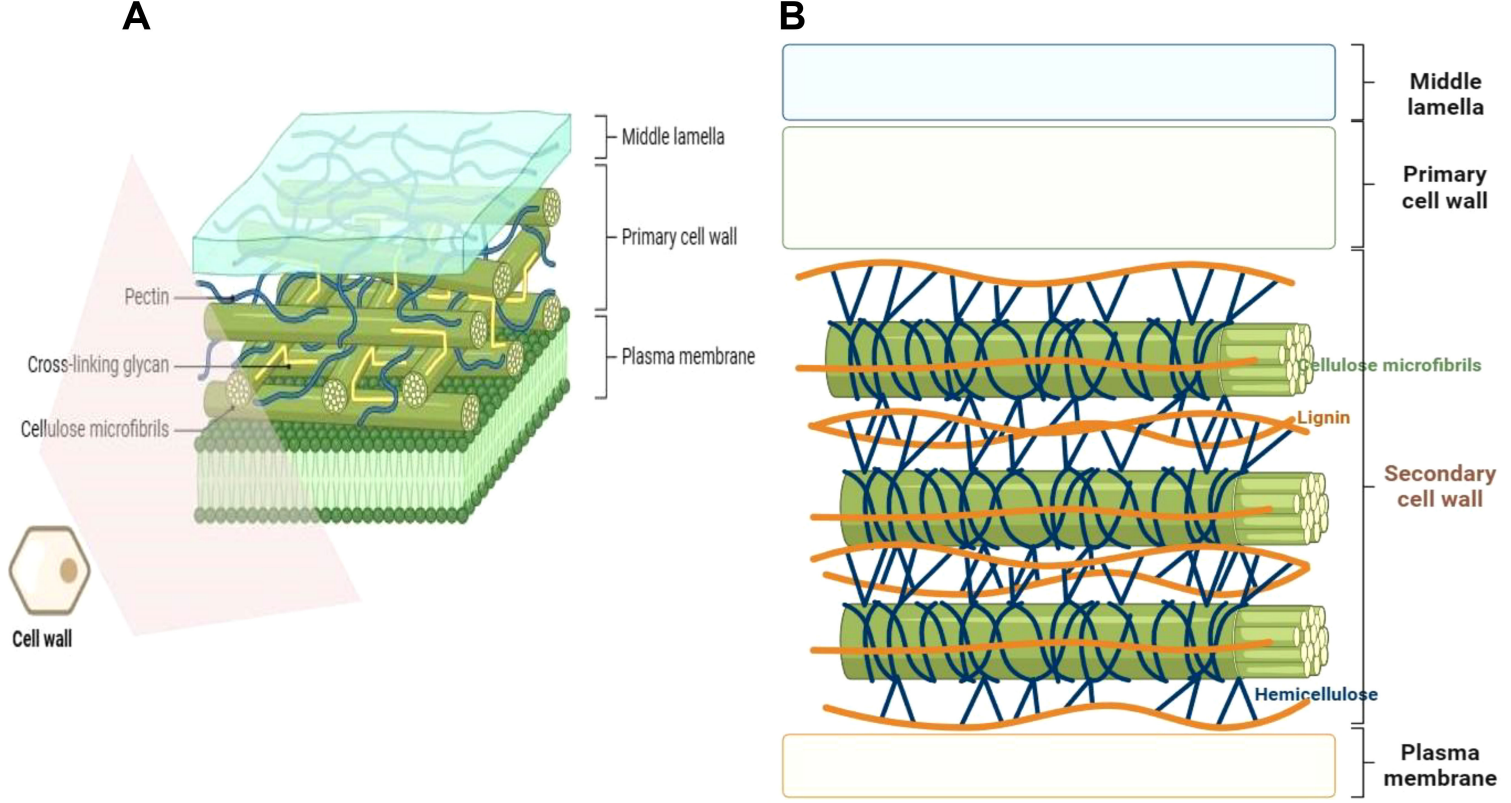
Plant cell wall position and structural configuration. **(A)** The primary cell wall is composed of cellulose microfibrils, pectin matrix, cross-linking xyloglucan hemicelluloses, and glycoproteins. It lacks lignin. **(B)** The secondary cell wall comprises of cellulose microfibrils and xylan hemicelluloses embedded with the hydrophobic polyphenol lignin and gylcoproteins. Some components like glycoproteins are not shown for simplicity purposes. The diagrams were created in Biorender.com (https://app.biorender.com/).

Plant cell wall is a complex assembly, primarily constituted by polysaccharide polymers (cellulose, hemicelluloses, and pectins), comprising distinct monosaccharides moieties joined by different type of bonds. The polymers may possess different biochemical decorations/modifications (such as methylations, acetylation, calcium bound ions) and may form connections with other cell wall components such as structural proteins (glycoproteins) and phenolic compounds (eg. polyphenolic lignin) (Cosgrove, 2005; Loqué et al., 2015; Höfte and Voxeur, 2017; Anderson and Kieber, 2020; Delmer et al., 2024). These different complex materials are woven into a strong, dynamically organized, and adaptable polymer structure primed for different functions (growth, pathogen defense, stress response, etc.) depending with the situation (Miedes et al., 2014; Wu et al., 2018; Zhang et al., 2021b). Although specific plant cell wall constituents and overall composition vary with plant species, tissue type, and tissue developmental state (Braidwood et al., 2014; Loqué et al., 2015), generally, polysaccharides constitute more than 80% of the total composite volume, with structural proteins and other minor components (eg., minerals) filling up the balance (Höfte and Voxeur, 2017). The plant cell wall is multi-layered or organized from the outside towards the plasma membrane (PM) as the middle lamella and primary cell wall (PCW), or/and secondary cell wall (SCW) in some species (Loqué et al., 2015; Höfte and Voxeur, 2017; Wu et al., 2018) (**Figure 1**). The dynamic PCW is formed in young dividing cells and functions to provide flexibility and basic structural support, protecting the cell and facilitating cell to cell interactions, whereas the thicker and more durable SCW is located between the PCW and the PM and is deposited beneath the primary wall of some specific cell types at a later stage after the cell has ceased growing and dividing (Hamann, 2012; Houston et al., 2016; Li Z. et al., 2016). The SCW is considered a vital adaptation characteristic that enables upright growth and environmental stress endurance in land plants (Houston et al., 2016).

Sandwiched between the middle lamella and the plasma membrane, the thin layered primary cell wall is built up of polysaccharides cellulose, hemicelluloses and pectin (**Figure 1A**). The PCW lacks lignification, and is pervious to small molecules. At the same time, it is elastic and extensible to enable the growth of the cell via the acid growth mechanism (Cosgrove, 2005; Braidwood et al., 2014; Dabravolski and Isayenkov, 2023). Depending with the relative amounts (composition) of different polysaccharides, the PCW can be categorized into type I and type II cell walls. Type I PCWs are found in dicots, non-commelinoid monocots and gymnosperms (such as conifers), whilst type II PCWs are limited to commelinoid monocots, including grasses (Carpita, 1996; Vogel, 2008; Sarkar et al., 2009; Barnes and Anderson, 2018). Grass cell wall type and dicots cell wall type also exhibit further differences in types of lignin and ferulic acid esterification they possess (Sarkar et al., 2009). Both type I and type II PCWs possess same amounts of cellulose, but differ in proportion of hemicelluloses (Carpita and McCann, 2020). Whereas type I PCWs contain a lesser proportion of hemicelluloses and a greater pectin content, there is predomination of hemicellulose but less pectin content in type II PCWs (Vogel, 2008; Barnes and Anderson, 2018). Additionally, type I PCWs have a greater proportion of structural proteins as compared to type II PCWs. Furthermore, the two PCW types have different hemicellulose composition, whereby type I PCW hemicellulose is mainly comprised of xyloglucan (XyG) and minor amounts of glucomannans, arabinoxylans and glucuronoxylans. Contrariwise, type II PCW hemicellulose largely contains glucuroarabinoxylans (GAX) and less of mixed-linkage β-(1,3)-(1,4)-_D_-glucan (MLG) and XyG. Besides, type II PCWs possess hydroxycinnamic acids that crosslink with GAX (Carpita, 1996; Vogel, 2008; Sarkar et al., 2009; Le Gall et al., 2015; Barnes and Anderson, 2018; Carpita and McCann, 2020).

The thick layered, stiff, and often waterproof, secondary cell wall forms beneath the PCW in some specific cell types, and is constituted by cellulose, xylan hemicelluloses and lignin (Kang et al., 2019; Dabravolski and Isayenkov, 2023) (**Figure 1B**). Lignin is a major structural component of plant secondary cell walls. It is a complex polymer composed of covalently joined monolignol subunits (coniferyl, sinapyl, and hydroxyphenyl alcohols) derived from amino acid L-phenylalanine linked via free radical coupling. The monolignol subunits are subjected to redox-mediated polymerization to produce guaiacyl (G), syringyl (S), hydroxyphenyl (H) units of lignin, respectively (Vanholme et al., 2010). Gymnosperm lignins are almost entirely made of G units, whilst angiosperm lignins are composed of mainly G and S units, with much reduced percentage of H units (Vanholme et al., 2010). Due to their assemblage by non-enzymatic polymerization, lignin chains lack a definite structure (Delmer et al., 2024). Lignin is sometimes covalently connected to ferulated xylan side chains, and renders apoplastic cell wall domains stiff/firm (Swaminathan et al., 2022). This increases the plant tissue`s structural robustness or mechanical strength (Polo et al., 2020), and facilitates its better resistance to pathogen attack and acclimatization to environmental changes (Khasin et al., 2021; Yadav and Chattopadhyay, 2023). SCWs of certain boundary tissue layers of plants such as root endodermis may be enriched with suberin, a lipophilic polymer composed of phenolic-derived glycerol, fatty acids, and aromatics, that provide structural support to these SCWs (Woolfson et al., 2022). Besides, suberin may act as a protective barrier, controlling water and ion transport (Vishwanath et al., 2015) (discussed in detail later under section 4.5).

Cellulose constitutes the major polymer in most plant cell walls, and is comprised of unbranched β -(1,4)-linked glucan chains (Zhang et al., 2021b). Despite the simplistic structural nature of cellulose, its bundling into multiscale cellulosic fibrils creates a complex nanostructure with high tensile strength and an important load-bearing function (Zhang et al., 2021b). Each cellulose fibril is composed of fundamental units called microfibrils, of approximately 35 Å width, corresponding roughly to a 6×6 array of chains. These native cellulose chains (cellulose I) are in a parallel arrangement as has been revealed by diffraction patterns analyses, high-resolution electron microscopy, and atomic force microscopy (AFM) (see (Delmer et al., 2024) and references therein). The microfibrils are modelled in a crystalline form, with the parallel chains oriented in cellulose Iα or Iβ lattices, with hydrogen-bonded sheets of chains running diagonally across the rectangular cross section (see (Jarvis, 2017)). These microfibrils are synthesized by the cellulose synthase (CeSA) enzyme complexes (Turner and Kumar, 2017; Wilson et al., 2021). The cellulose synthase complexes are thought to be assembled in the Golgi apparatus and transported to the PM via vesicle trafficking (Zhang et al., 2021b).

Hemicelluloses comprise of xylans, xyloglucans, β-(1,3;1,4)-glucan, mannans, and glucomannans. They all contain β-(1,4)-glycosyl connected backbones with the same equatorial arrangements (Zhang et al., 2021b). Except for xylans (whose backbone is synthesized by the GT47 and GT43 family Type II membrane proteins (Wu et al., 2010)), backbones of most hemicelluloses are synthesized by the GT2 family cellulose synthase-like proteins (Scheller and Ulvskov, 2010). However, their glycosyl residues vary. The backbones are frequently replaced by different glycosyl residues with unique patterns, which explains their architectural and physiochemical differences (reviewed in (Zhang et al., 2021b)). Xylan is the most abundant hemicellulosic polysaccharide in vascular plants (most common in type II primary cell walls and eudicot secondary walls), with a β-(1,4)-linked xylosyl sugar backbone decked with non-/methylated or non-/feruloylated arabinose and glucuronic acid residues as side chains. Xyloglucan hemicellulose is the principal polysaccharide in type I (dicotyledonous) primary walls, possessing a β-(1,4)-linked glucan sugar backbone decked with side chains of xylose, fucose, and galactose residues (Zabotina, 2012). Other hemicelluloses include mannans and glucomannans. Mannans are commonly found in gymnosperms, and possess linear glycan chains with a β-(1,4)-linked mannose backbone. Glucomannan is just mannan with intercalary β-(1,4)-linked glucose in its backbone (Carpita and Gibeaut, 1993; Ebringerová, 2005; Anderson and Kieber, 2020).

Pectin is a highly complex and heterogeneous polysaccharide, with d-galacturonic acid (GalA) residues linked via α-1,4-glycosidic bonds (Mohnen, 2008). Pectin is synthesized in the Golgi apparatus in methylesterifed form (with homologalacturonan methylesterified at the C-6 position) and exported into the wall where it is demethylesterified by the action of PMEs (Pelloux et al., 2007; Mohnen, 2008; Harholt et al., 2010; Wu et al., 2022). Pectin is composed of four main types of domains, viz., homogalacturonan (HG), rhamnogalacturonan I (RGI), RGII, and xylogalacturonan (XGA), covalently linked to create a pectin matrix (Willats et al., 2001; Mohnen, 2008; Ropartz and Ralet, 2020). HG, or the pectin smooth region, is the dominant component of pectin, and comprises α-1,4-linked d-GalA chains, and contributes to structural elasticity (Ridley et al., 2001; Willats et al., 2001; Ropartz and Ralet, 2020). HG is synthesized by galacturonosyl transferases (GAUTs) of the GT8 family (Atmodjo et al., 2013). PME can act upon HG, in both random and blockwise demethylesterification, thus playing dual roles in both cell wall loosening and stiffening (Du et al., 2022). In the former, HG can become susceptible to the actions of other cell wall degrading enzymes such as polygalacturonases and pectate lyases, resulting in cell wall loosening. In the later, HG demethylesterified in a blockwise fashion exposes carboxyl groups which can cross-link with Ca^2+^ ions to form a pectate gel network, known as an “egg box”, which contributes to cell wall stiffening (Pelloux et al., 2007). RGI backbone is built upon galacturonic acid and rhamnose residues, laced with arabinan, arabinogalactan, and galactan side chains; whereas RGII comprises of HG backbone decked with side chains of several sugar units and diverse glycosyl linkages (Anderson and Kieber, 2020; Malacarne et al., 2024). Pectin makes up the main constituent of primary cell walls, in both dicotyledonous (~ 35%) and monocotyledonous (2-10% in Gramineae) species, playing essential roles in cell adhesion and cell wall plasticity (O’neill et al., 1990; Willats et al., 2001; Mohnen, 2008; Wu et al., 2018). Thus, pectins control cell and organ growth, development, cell-wall porosity, and response to environmental cues (Willats et al., 2001; Caffall and Mohnen, 2009; Wolf and Greiner, 2012; Wu et al., 2022). Despite also being localized in the PCW and SCW, pectins are more enriched in the middle lamella, and their biosynthesis requires different kinds of transferases, such as glycosyl-, methyl-, and acetyltransferases (Ridley et al., 2001; Mohnen, 2008; Caffall and Mohnen, 2009; Yang and Anderson, 2020).

Besides, cell wall-related proteins and enzymes, including expansins (α-expansins, β-expansins, EXPLA, EXPLB), xyloglucan endo-β-transglucosylases/hydrolases (XET/XTHs), endo-1,4-β-D-glucanase (EGase), extensins, proline rich proteins and glycine-rich proteins, as well as minor components (eg., minerals, small metabolites, etc.) are also important constituents of cell walls (Jamet et al., 2006; Qiu et al., 2021). Cell wall-related proteins regulate cell wall extensibility, which modulates cell enlargement and expansion (Le Gall et al., 2015). Expansins induce wall creep and wall relaxation, via loosening of the linkages between cellulose microfibrils (Cosgrove, 2000; Sampedro and Cosgrove, 2005; Cosgrove, 2016b) (discussed in detail hereafter in section 4.2.1). Extensins (eg. leucine-rich repeat extensins) and hydroxyproline-rich proteins (HRGPs) are involved in regulating cell wall expansion, cell growth, and cell wall integrity sensing (Herger et al., 2019). Other cell wall-modifying enzymes crucially regulate cell wall plasticity/rheology (Cosgrove, 1999; Le Gall et al., 2015). These cell wall-modifying proteins actively participate in cell wall remodeling processes in response to different growth and stress stimuli (Höfte and Voxeur, 2017; Anderson and Kieber, 2020). In summary, plant cell wall is a complex and highly dynamic entity whose components and structure are constantly and appropriately modified during growth and development and in response to environmental cues.

## 3 Molecular and genetic regulation of cell wall related sensing and signaling systems involved in abiotic stress response

Plant cells possess an effective CWI sensing mechanism that monitors functional and structural changes to the cell wall to ensure a balance between cell wall biosynthesis and turgor-driven cell wall extension/growth without compromising the CWI (Vaahtera et al., 2019; Rui and Dinneny, 2020). Most of the work on CWI sensing in plants is based on *Arabidopsis thaliana* (Arabidopsis), but its elucidation in other species is gaining traction. CWI signaling pathway is initiated by PM-localized cell surface sensors, including several members of the receptor-like Ser/Thr protein-kinase (RLK) and receptor-like protein (RLP) families, and relayed downstream via the mitogen-activated protein kinase (MAPK) cascades among other signaling modules (Bashline et al., 2014; He et al., 2018). The molecular mechanisms of CWI-modulated stress signaling share commonalities with the osmotic signaling cascade, that may be drought- or salinity-induced. For instance, alterations in cell water potential and turgor pressure are common among these stresses (Verslues et al., 2006; Novaković et al., 2018). In fully hydrated (> 60%) primary cell walls, reduced cell water status alters the gel-like matrix, which affects the organization and interaction of cell wall polymers and the wall-plasma membrane physical connection (Thompson and Islam, 2021). In ion toxicities, for instance high salinity-induced, monovalent ions (eg., Na^+^ and K^+^) may displace Ca^2+^ ions and disrupt the pectin “egg box” matrix structure (Peaucelle et al., 2012; Engelsdorf et al., 2018). These stress-induced alterations in cell wall mechanical properties yield CWI changes that are perceived by the CWI sensors such as Wall Associated Kinases (WAKs), Feronia (FER), and Receptor-Like Protein Kinases (RPKs) (Ringli, 2010; Hamann, 2012; Novaković et al., 2018; Vaahtera et al., 2019; Liu J. et al., 2021; Baez et al., 2022). Meanwhile, cold stress may cause the ice crystallization of the apoplastic space, which may result in cell wall deformation (Rajashekar and Lafta, 1996). Cold and drought stresses may similarly affect cellular exchanges through a decrease in membrane permeability or a decrease in water content and plasmolysis. Osmotic adjustment becomes critical in response to both stresses to ensure osmotic potential and a protective layer around cell structures and macromolecules (Charrier, 2021). Thus, plants have developed similar molecular response pathways for these stresses, mediated, in part, by abscisic acid (ABA) and Dehydration-Responsive Element (DRE) *cis*-acting element or C-Repeat (CRT) (Yamaguchi-Shinozaki and Shinozaki, 1994; Stockinger et al., 1997; Nakashima et al., 2014; Charrier, 2021; Kim et al., 2024); these pathways regulate responses to osmotic stress (Charrier, 2021). Besides, both drought and cold stresses induce stomatal closure, although, in cold stress, this mechanism seems to be ABA-independent (Wilkinson et al., 2001; Kim et al., 2024). It will be crucial to understand how these combined stresses are perceived by the cell wall and get integrated to the CWI maintenance pathway to produce a robust stress response. This will create a possibility to enhance plant drought tolerance via cell wall modification (Barbut et al., 2024).

**Figure 2:**
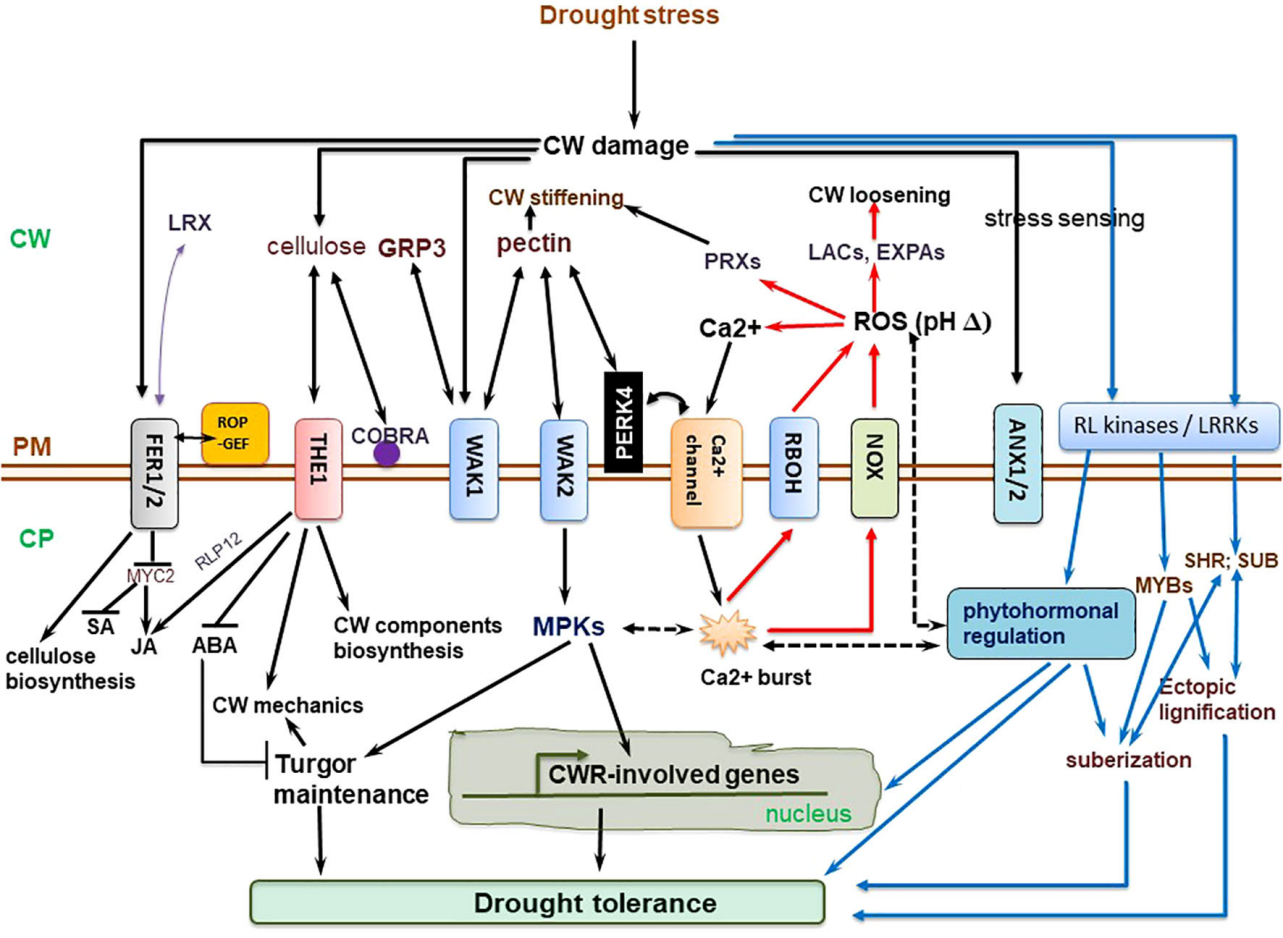
Molecular regulation mechanisms of cell wall (CW) remodeling related to plant drought tolerance. Receptor-like kinases (RLKs) such as leucine-rich repeat receptor-like kinases (LRRKs), wall-associated kinases (WAKs), and extracellular proteins, including extracellular leucine-rich repeat-containing extension 1(LRX1) perceive drought-triggered CW damage or composition and/or structural alterations to orchestrate CW stress signaling, which is mediated via mitogen-activated protein kinases (MAPKs). The MAPK cascade regulate turgor maintenance through vacuolar invertase, and together with NACs and MYB transcription factors, regulate the activation of downstream CW remodeling-involved genes. The CWI maintenance pathway crosstalk with ROS and phytohormonal signaling pathways to actuate plant drought tolerance. Plasma membrane (PM)-localized proline-rich extension-like receptor kinase 4 (PERK4) interacts with pectin and stimulates Ca^2+^ channels, resulting in cytosolic Ca^2+^ burst, which alters intracellular and extracellular pH, and trigger NADPH oxidase (NOX)-reliant reactive oxygen species (ROS) accumulation. The Ca^2+^ influx evokes respiratory burst oxidase homologues, causing ROS accumulation in the cell wall. This, together with peroxidase (PRX) activity, facilitates oxidative crosslinking of extensins and pectin accumulation, stiffening the cell wall. On the other hand, limited PRX activity or H_2_O_2_ generation trigger OH•− radicals accumulation, increased severing of sugar bonds in polysaccharides, and eventual CW loosening. THESEUS1 (THE1) perceives cellulose reduction-induced structural defects in CW, whereas COBRA is a PM-localized and GPI-anchored protein crucial for cellulose microfibrils alignment. ANX1/2, ANXUR1/2 (closest homologs of FER in *Arabidopsis thaliana*) modulate pollen tube rupture which encompass rapid alterations in CW composition and architecture (Ringli, 2010). Complete black arrows denote cell wall integrity (CWI) maintenance signaling/mechanisms, blue lines relate to phytohormonal regulation and lignification mechanisms already discussed in detail in other sections, red lines relate to reactive oxygen species (ROS) signaling, whereas black dotted lines imply crosstalk among these pathways. ABA, abscisic acid; CP, cytoplasm; EXPAs, expansins; FER1/2, FERONIA 1/2; GRP3, Glycine-rich protein 3; LACs, laccases; JA, jasmonic acid; ROP-GEF, Rho-of-plants-guanosine nucleotide exchange factors; SA, salicylic acid; SHR, SHORT-ROOT transcription factor; SUB, SUBERMAN transcription factor. The illustration is based on (Ringli, 2010; Novaković et al., 2018; Gigli-Bisceglia et al., 2020; Bacete et al., 2022) and others discussed in text.

The cell wall-perceived stress signal is transduced into the cell, prompting an eventual repertoire of responses to be coordinated (Ringli, 2010; Smokvarska et al., 2020). Cell wall-mediated stress responses encompass CWI sensing, ROS generation, and phytohormonal signaling pathways converging (Rao and Dixon, 2017; Novaković et al., 2018; Vaahtera et al., 2019) (**Figure 2**). Hyperosmotic stress triggers ROS accumulation, which act as secondary messengers for inducting several plant responses (Martinière et al., 2019). Ca^2+^ influx evokes Respiratory Burst Oxidase Homologues (RBOHs), triggering the production of ROS (H_2_O_2_ and OH•−) in the cell wall (Suzuki et al., 2011). The osmotically triggered ROS buildup requires Rho-of-plants 6 (ROP6), an upstream activator of RBOH D or/and F (Martinière et al., 2019). The ROP6 creates osmotic stimuli-dependent nanodomains within the plasma membrane, ensuring signal specificity (Smokvarska et al., 2020). When combined with POD activity, ROS production fuels radical coupling of extensins and signaling, resulting in pectate buildup and wall stiffening (Francoz et al., 2015) (**Figure 2**). However, limited POD activity or H_2_O_2_ induces OH•− radicals’ formation, triggering the severing of polycarbohydrate sugar bonds, and consequent cell wall loosening (Kidwai et al., 2020).

Several receptor-like kinases, including WAKs family, *Catharanthus roseus* Receptor-Like Kinase1-Like (*CrRLK1L*), FER, Leucine-Rich Repeat Receptor-Like Kinases/Protein Kinases, etc. have been known to mediate cell wall stress sensing and signaling (Ringli, 2010; Steinwand and Kieber, 2010; Wolf and Greiner, 2012; Osakabe et al., 2013; Galindo-Trigo et al., 2016; Wolf, 2022). WAKs are the most studied RLKs and are highly conserved in Arabidopsis in five transmembrane protein families (Steinwand and Kieber, 2010). They harbor a cytoplasmic serine threonine kinase, a transmembrane domain, and an extracellular domain (Kohorn and Kohorn, 2012). WAKs, such as WAK1, seem to directly bind to polysaccharides in the wall, through their extracellular N terminus firmly attaching to Ca^2+^ cross-linked pectin-derived oligogalacturonides (He et al., 1996; Decreux and Messiaen, 2005), to initiate cell wall perception and signaling through MAPKs (such as MAPK3, MAPK6, etc.) and modulate vacuolar invertases and turgor maintenance (Kohorn et al., 2006; Kohorn and Kohorn, 2012). Meanwhile, *WAK1* has also been shown to interact with the glycine-rich protein *AtGRP-3*, which is a cell wall-localized structural protein (Park et al., 2001). WAKs are involved in cell expansion (Wagner and Kohorn, 2001), and are also induced by, and participate in the response to, pathogen attack and several stresses such as wounding, heavy metals, etc. (reviewed in (Ringli, 2010; Kohorn and Kohorn, 2012)). Disruption of *WAK* expression using *WAK2* antisense RNA led to reduced leaf cell expansion (Wagner and Kohorn, 2001), whereas *wak2* loss-of-function mutants and *WAK4* antisense-RNA-expressing seedlings showed impaired root cell elongation (Kohorn et al., 2006). The growth performance of *wak2* loss-of-function mutants exhibited a dependence on extrinsic sugars, signifying that possibly WAKs provide a cell-wall-sensing function, mediated by pectins and sugar metabolism (Kohorn et al., 2006; Kohorn and Kohorn, 2012).

Other members of the RLK family also participate in CWI sensing. Theseus1 (THE1), a member of the *CrRLK1L* family, was identified as a suppressor of the short-hypocotyl phenotype in the cellulose-deficient *procuste1-1* (*pcr1-1*) mutant (Hématy et al., 2007).Under control (non-stress) conditions, knockout mutants (*the1-1*, *the1-2*, *the1-3* and *the1-6*) did not exhibit any phenotypic defects, signifying that THE1 is only activated upon CWI being compromised, reinforcing the idea that THEI functions as a CWI sensor (Hématy et al., 2007; Baez et al., 2022). Additionally, *the1* attenuated hypocotyl growth inhibition in other cellulose-deficient mutants, such as *cesa3eli1*, *cesa1rsw1*, etc (Hématy et al., 2007; Hématy and Höfte, 2008), suggesting that THE1 is activated by cellulose synthesis perturbation, and may act as a CWI sensor, which in turn evoke the expression of downstream candidate genes that regulate cell elongation (Bashline et al., 2014; Novaković et al., 2018). Meanwhile, Feronia (FER) is a PM-localized receptor kinase and most characterized CWI sensor from the *CrRLK1* family (Galindo-Trigo et al., 2016). FER synergizes with different Rapid Alkalinization Factor (RALF) peptide ligands to function in several growth, development, and stress response processes, including CWI maintenance in growing-tip or elongating root-tip cells (Duan et al., 2010; Stegmann et al., 2017; Feng et al., 2018; Cheung, 2024). FER acts as a receptor for numerous RALFs, including RALF1 and RALF34 (Li C. et al., 2016; Gonneau et al., 2018). FER-RALF1 interaction enhances FER phosphorylation ability but inhibits across PM proton transport directed by H^+^-ATPase (Li C. et al., 2016), which possibly influences cell wall remodeling according to the acid growth theory (Haruta et al., 2014; Baez et al., 2022). FER and its RLK relatives possess extracellular domains that interact with cell wall carbohydrate moieties to sense cell wall perturbations and initiate appropriate cellular responses (Li C. et al., 2016; Moussu et al., 2023). For example, the FER extracellular domain directly interacts with pectin to sense salinity-induced wall defects, and trigger corresponding stress responses (Feng et al., 2018). In addition to the PM and cell wall, the RALF-FER signaling cascades interact with molecules in the cytoplasm and nucleus to modulate a complex intertwined signaling network (Cheung, 2024). FER also interacts with ROP-GEF [Rho-of-plants (ROP)-guanosine nucleotide exchange factors (GEF)] to facilitate the ROP2 exchange of GDP to GTP (Igisch et al., 2022), RBOH activation, and ROS production (Galindo-Trigo et al., 2016; Novaković et al., 2018). Disruption of FER function decreases ROPs levels, hinders ROP-mediated and RBOH-reliant ROS synthesis (Duan et al., 2010). More recently, it has been shown that FER controls the accrual and nano-scale compartmentalization of phosphatidylserine in the PM, to regulate Rho GTPase signaling in Arabidopsis (Smokvarska et al., 2023). Overall, considering the functional diversity of FER and the mechanistic complexity of the FER-anchored signaling modules, FER provides a rich ground for research that could help uncover new insights on plant abiotic stress response and signaling (Li C. et al., 2016; Cheung, 2024).

FEI1 and FEI2, leucine-rich repeat RLKs, belong to RLK subfamily XIII, which is different from the WAK and THE1 subfamilies (Xu et al., 2008; Bashline et al., 2014). They are important for the non-uniform expansion of different root cells in Arabidopsis, as well as cell extension in Arabidopsis stamen (filaments) and etiolated seedling hypocotyls (Xu et al., 2008). *FEI1* and *FEI2* gene mutations interrupt the non-uniform (anisotropic) cell expansion, hinders biosynthesis of wall polymers, and fortify cellulose biosynthesis repressors (Xu et al., 2008). The *fei1 fei2* roots with expansion defects were rescued by the disruption of only 1-aminocyclopropane-1-carboxylic acid (ACC) synthase (an ethylene biosynthesis-related enzyme involved in conversion of Ado-Met to ACC), and not the entire ethylene (Et) response pathway, suggesting that FEI kinases crucially mediate a signaling pathway that integrates cell wall biosynthesis and ACC synthase in Arabidopsis (Xu et al., 2008). More recently, *FEI1*, *FEI2*, and *Altered Root Hydrotropic Response 1* (*ARH1*), the three closely linked *RLKs*, have been shown to exhibit polar localization at the PM regions of Arabidopsis root tips (Chang et al., 2024). Overexpression of these three genes greatly reduced root hydrotropism, but their corresponding loss-of function mutants showed an increased root hydrotropic response tips (Chang et al., 2024). Additionally, the triple mutant *arh1-2 fei1-C fei2-C* showed cell wall, cutin, and wax (CCW) biosynthesis impairments in its root tips, suggesting that the root tip cell wall integrity, cutin and wax status mediate a balance between root hydrotropism and osmotic tolerance (Chang et al., 2024); this will need further exploration as it may also crucially regulate root responses to drought stress.

A PM-localized receptor-like protein, RLP44, mediates the response to pectin modification through activation of brassinosteroid (BR) signaling pathway (Wolf et al., 2014). RLP44 mediates this activation via direct connection with the BR co-receptor *BAK1* (*Brassinosteroid-Insensitive 1(BRI1)-Associated Receptor Kinase 1*), to integrate cell wall surveillance with hormone signaling, and regulate CWI sensing and growth in Arabidopsis (Wolf et al., 2014). *BAK1* can be activated upon both abiotic and biotic stresses, and can also act as a co-receptor for several RLPs mediating DAMP (Damage-Associated Molecular Patterns) and PAMP (Pathogen-Associated Molecular Patterns) recognition (Yasuda et al., 2017; Novaković et al., 2018; Molina et al., 2024a). DAMPs and PAMPs are unique CWI sensors that detect plant cell wall damage (eg. to cellulose and other polysaccharide components such as pectins) caused by pathogen infection, wounding, or other stresses, to activate RLKs or receptor kinases (RKs) that initiate stress signaling cascades (for reviews, see (Molina et al., 2024a; Molina et al., 2024b)). At the same time, these stress-induced cell wall damages prompt cell wall remodeling to ensure CWI maintenance (Hématy et al., 2007; Novaković et al., 2018). In case of DAMPs, the caused by these stresses leads to the release of carbohydrate-based wall molecules (glycans) that are recognized/perceived by the extracellular ectodomains (ECDs) of pattern recognition receptors (PRRs) as DAMPs to actuate pattern-triggered immunity (PTI) response and disease resistance (Molina et al., 2024b). For PAMPs, specific ECD-PRRs of RKs (eg., RKs with leucine-rich repeat and Malectin domains within their ECDs, LRR-MAL RKs (Martín-Dacal et al., 2023)) or RLPs (eg., lysine motif, Lys-M *OsCERK1* (Shimizu et al., 2010)) can recognize oligosaccharide/polysaccharide molecules emanating from pathogens in the apoplast as PAMPs (Novaković et al., 2018; Molina et al., 2024b). Examples of PAMPs include chitooligosaccharides from fungi, flagellin from bacterial flagellae, etc (Boller and Felix, 2009; Novaković et al., 2018; Molina et al., 2024b). Likewise, specific ECD-PRRs recognize glycans emanating from the walls and extracellular layers of microbes interacting with plants as microbe-associated molecular patterns (MAMPs), evoking corresponding PTI responses (Boller and Felix, 2009; Molina et al., 2024a). Despite not directly linked to abiotic stress response, further elucidation of these unique glycan-based recognition mechanisms may reveal any possible similarities with abiotic-stress-specific cell wall sensors, which could help in identifying novel abiotic stress cell wall sensors in plants.

Meanwhile, another key receptor, Arabidopsis Receptor Like Protein Kinase 1 (RPK1), is only responsive to abiotic stress, and its overexpression enhances tolerance to drought, heat, salinity, and cold stresses (Osakabe et al., 2010; Novaković et al., 2018). Other RLKs involved in cell wall sensing, including PERK4 (Proline-rich Extension-like Receptor Kinase 4), are discussed in recent reviews ( (Baez et al., 2022; Wolf, 2022). The exact functions of these several RLKs and RLPs are yet to be elucidated. Nonetheless, we expect that as research on CWI sensing and stress signaling gathers much pace, new details will emerge that will help us assign receptor/gene functions and clarify the complex signaling crosstalk involved in abiotic stress response (Novaković et al., 2018). Downstream of these receptor complexes, MAPK cascades are among the key signaling modules which integrate signals and regulate diverse cellular and physiological responses via phosphorylation of several downstream targets (reviewed in (He et al., 2018)).

NAC [no apical meristem (NAM), Arabidopsis ATAF1/2, and CUC2 (cup-shaped cotyledon)] and MYB (myeloblastosis) TFs crucially modulate the induction of downstream SCW-biosynthetic or stress-responsive genes (Wang and Dixon, 2012; Cao Y. et al., 2020), with the transcriptional regulation exhibiting a high plasticity to abiotic stress, which helps plants to appropriately adapt to the stress (Nakano et al., 2015; Yoon et al., 2015; Houston et al., 2016; Choi et al., 2023). For instance, transcriptional actuation of SCW-biosynthesis-related genes by rice and maize secondary wall NACs (SWNs, that is, *OsSWNs* and *ZmSWNs*) rescued an Arabidopsis *snd1 nst1* double mutant that had secondary wall thickening defects (Zhong et al., 2011). Overexpressed *OsSWNs* and *ZmSWNs* significantly activated several SCW-related TFs and biosynthetic genes in Arabidopsis, simultaneously increasing cellulose, xylan, and lignin accrual (Zhong et al., 2011). Additionally, *OsMYB46* and *ZmMYB46*, the functional orthologs of *AtMYB46/AtMYB83*, effectively activated the whole SCW biosynthesis system after overexpression in Arabidopsis. *OsSWN*s and *ZmSWN*s activated *OsMYB46* and *ZmMYB46* by directly binding to the SCW NAC-binding elements (SNBEs) at their (*OsMYB46* and *ZmMYB46*) promoters (Zhong et al., 2011). Reasonably, these NAC and MYB TFs could be candidates for manipulation to influence lignin biosynthetic genes expression changes that possibly modify plant cell walls, via increased lignin accumulation, to enhance plant drought tolerance (Miyamoto et al., 2020; Han et al., 2022).

Recently, SHORT-ROOT (SHR) transcription factor, a master regulator of endodermal development, has been observed to mediate a transcriptional interplay between lignification and suberization, integrated to stress signaling (Xu H. et al., 2022). Additionally, 13 key MYB TFs (including *MYB74*, *MYB68*, *MYB36*, *MYB122*, *MYB41*, *MYB39*, *MYB52*, *MYB53*, etc.) that form multiple sub-networks mediating feedback or feed-forward loops to balance this interplay were uncovered (Xu H. et al., 2022). Among them, sub-networks involving nine MYB TFs were shown to interact with ABA signaling to integrate stress response and root development, suggesting that SHR and these key MYB TFs crucially modulate and integrate multiple developmental and stress signals, and are key targets for genetic engineering for enhancing plant stress adaptation (Xu H. et al., 2022). Equally, the function of SUB TF (partly discussed in section 3 above) crucially modulates transcriptional networks related to suberin, lignin, and phenylpropanoid biosynthesis, as well as phytohormonal signaling (Cohen et al., 2020); hence, its characterization and potential targeting for genetic engineering may be a key step towards enhancing cell wall remodeling-mediated drought tolerance in crop plants (Cohen et al., 2020).

*Photo-sensitive Leaf Rolling 1* (*PSL1*) gene encrypts a cell wall-localized polygalacturonase (PG) that alters cell wall structure and enhances rice drought tolerance (Zhang G. et al., 2021). A *psl1* mutant, exhibiting ‘napping’ phenotype and reduced growth, displayed significant cell wall composition modifications as compared Wt plants (Zhang G. et al., 2021). Such cell wall composition alterations improved the mutant`s drought tolerance, through decreasing osmotic and drought stress-induced water loss. Collectively, these results suggested that *PSL1*, acting as PG, modifies cell wall biosynthesis, plant development, and enhances rice drought stress tolerance (Zhang G. et al., 2021). Equally, *Curled Leaf and Dwarf 1*/*Semi-Rolled Leaf 1 (CLD1*/*SRL1*) gene, that encrypts a glycophosphatidylinositol (GPI)-anchored protein (GAP) involved in controlling other growth and development facets in rice, functionally regulates rice leaf rolling by influencing cell wall synthesis, epidermis integrity, and water homeostasis (Li et al., 2017). A *cld1* mutant shows substantially decreased cellulose and lignin contents in leaf SCWs, signifying that deterred *CLD1/SRL1* function impacts cell wall development (Li et al., 2017). Additionally, *CLD1/SRL1* function deficiency results in leaf epidermis defects (eg., formation of bulliform-like epidermal cells), which reduce the water retention capacity and causes water deficit in *cld1* mutant leaves – the main contributor to leaf rolling. Due to the accelerated leaf water loss and reduced leaf water content, *cld1* mutant shows decreased water deficit stress tolerance (Li et al., 2017). Overall, *CLD1/SRL1* may play an essential role in improving plant drought tolerance, by functionally regulating leaf-rolling and minimizing leaf transpiration (Li et al., 2017), and thus, can be targeted for genetic engineering to enhance crop drought tolerance.

Cellulose synthase gene *AtCesA8/IRX1* participates in SCW synthesis, and influences plant drought and osmotic stress tolerance (Chen et al., 2005). Disruption of *AtCesA8/IRX1*, combined with other two allelic mutants, *leaf wilting 2-1* (*lew2-1*) and *lew2-2*, improved drought and osmotic stress tolerances in mutant plants as compared to Wt Arabidopsis (Chen et al., 2005). The *lew2* mutant accrued greater ABA, proline, and soluble sugar contents in comparison to Wt plants, revealing that knocking down of *LEW2* enhances drought tolerance, whereas cellulose synthesis is essential for plant response to osmotic and drought stresses (Chen et al., 2005). Other cell wall remodeling-involved genes important for improving plant drought tolerance are provided in **Table 2**. Leveraging on modern genomics tools such as genome-wide association studies, (GWAS), which now permit high-resolution mapping of QTLs, we can exploit the abundant natural genetic variation present in progenitor species (Bahri et al., 2020) and cultivars (Martínez et al., 2007), to identify and harness desirable cell wall properties for the genetic improvement of drought tolerance in crop plants (Yoshida et al., 2021).

**Table 2:** Selected cell-wall-modification-related gene candidates for enhancing drought tolerance in plants.

Host species	Gene name	Function	Strategy	Observed phenotype/outcome	References
*Malus domestica*	*MdMRLK2*	A FERONIA receptor kinase involved in plant growth, development and stress response	Overexpression	Enhanced energy levels, free amino acids, ABA content, and drought tolerance in overexpressing transgenic apple plants.	(Jing et al., 2023)
*Arabidopsis thaliana* (Arabidopsis)	*THESEUS1* (*THE1*)	A *CrRLK1L* RPK cell wall integrity sensor, required for hypocotyl cell elongation, responses to cell wall damage induced by cellulose biosynthesis inhibition and by pathogen infection.	Mutations and GFP fusion OE analyses experiments.	Mutations in THE1 and overexpression of THEI respectively attenuated and enhanced growth inhibition and ectopic lignification in seedlings mutated in cellulose synthase *CESA6* without influencing the cellulose deficiency.	(Hématy et al., 2007)
Arabidopsis	*THE1*	A *CrRLK1L* RPK cell wall integrity sensor, required for hypocotyl cell elongation, responses to cell wall damage induced by cellulose biosynthesis inhibition and by pathogen infection (Hématy et al., 2007; Engelsdorf et al., 2018)	Seedlings treated with cellulose biosynthesis inhibitor isoxaben (ISX), and turgor pressure influencer sorbitol, and effects visualized using Brillouin microscopy.	THEI modulated changes in turgor pressure and cell wall stiffness in Arabidopsis roots via ABA biosynthesis.	(Bacete et al., 2022)
Arabidopsis	*RPK1*	A PM-localized receptor-like protein kinase that functions as a regulator of ABA signaling in Arabidopsis.	Gene characterization, expression analyses, and several abiotic stress treatments analyses.	*RPK1* was rapidly induced by ABA, dehydration, salt and cold treatments, suggesting its role in abiotic stress response.	(Hong et al., 1997)
Arabidopsis	*RPK1*	A PM-localized receptor-like protein kinase that functions as a regulator of ABA signaling in Arabidopsis.	Disruption and overexpression experiments.	Transgenics overexpressing *RPK1* protein showed increased ABA sensitivity in their root growth, stomatal closure, less transpirational water loss, and enhanced drought tolerance. OE plants also showed enhanced oxidative stress tolerance.	(Osakabe et al., 2010)
Arabidopsis	*AtCesA8/IRX1* *(lew2-1 and lew2-2 )*	Involved in cellulose synthesis	Mutations/disruption	Mutants were more tolerant to drought and osmotic stresses. Higher accumulation of ABA and osmolites in *lew2* mutants than Wt. Higher expression of stress-inducible marker gene *RD29A*, proline synthesis-related gene *P5CS*, and an ABA synthesis-related gene *SDR1* in *lew2* than in Wt.	(Chen et al., 2005)
Tobacco (*Nicotiana tabacum*)	*TaEXPB23*	Wheat β-expansin gene	Overexpression under the control of a 35S promoter	*TaEXPB23*-OE tobacco showed slower water loss rate, and their cells maintained their structural integrity better than Wt under drought. Further, OE lines had higher photosynthetic performance and lower electrolyte leakage than Wt.	(Li et al., 2011)
Tobacco	*TaEXPB23*	Wheat β-expansin gene	Overexpression under the root-specific promoter PYK10.	*P10::TaEXPB23* plants showed an increase in lateral roots, improved water status, higher photosynthetic performance, reduced ROS, and enhanced drought tolerance than *35S::TaEXPB23* and WT plants.	(Li et al., 2015)
Tobacco	*TaEXPB23*	Wheat β-expansin gene	Overexpression	Increased the activity of CW-bound PRX and enhanced oxidative stress tolerance in transgenic tobacco.	(Han et al., 2015)
Rice (*Oryza sativa*)	*OsEXPA8*	Root-specific gene involved in cell extension	Overexpression	Enhanced rice growth and root system architecture	(Ma et al., 2013)
Tobacco	*TaEXPA2*	Involved in cell wall expansion control	Constitutive expression	Enhanced osmotic adjustment, antioxidant capacity, seed production, and drought tolerance in transgenic plants.	(Chen et al., 2016)
Arabidopsis	*AnEXPA1* and *AnEXPA2*	Regulation of cell wall loosening	Overexpression	Enhanced drought tolerance and survival rate in OE transgenic plants,	(Liu et al., 2019)
Soybean (*Glycine max*)	*GmEXPB2*	β-expansin gene	Overexpression and mutant analyses	*GmEXPB2* expression was induced by mild water deficiency and enhanced root drought tolerance in overexpressing plants.	(Guo et al., 2011)
Roses (*Rosa hybrida*) and Arabidopsis	*RhNAC 2 and RhEXPA4*	Involved in dehydration tolerance regulation during rose petal extension	Silencing of *NAC2* and *EXPA4*; Overexpression *of EXPA4*	Silencing of the two genes reduced the recovery of intact petals or petal discs during rehydration; Overexpression of *RhNAC2* and *RhEXPA4* conferred strong drought tolerance in transgenic Arabidopsis, and 20 CW-related genes were up-regulated in *RhNAC2*-OE Arabidopsis.	(Dai et al., 2012)
Maize	Cinnamoyl-CoA reductase 1, and 2	Involved in lignin biosynthesis	Up-regulated expression	Increased root lignification	(Fan et al., 2006)
Cluster bean	*PAL1, C4H, 4CL, CCR, CCOMT*, and *CAD6*	Involved in lignin biosynthesis	Up-regulated expression	Increased lignin synthesis, water-flow resistance, sustained nutrient transport, and enhanced drought tolerance.	(Narayan et al., 2023)
Rice	*CLD1/SRL1*	Encodes a glycophosphatidylinositol (GPI)-anchored membrane protein that modulates leaf rolling and other aspects of growth and development. Facilitates plasma membrane – cell wall communication.	Mutation analysis	*cld1* mutant showed significant decreases in cellulose and lignin contents in leaf secondary CWs; loss of function of *CLD1/SRL1* affected CW formation. Additionally, *cdl1* exhibited decreased water-retaining capacity, more rapid leaf water loss, lower leaf RWC, and reduced drought tolerance.	(Li et al., 2017)
Arabidopsis	*IRX14/IRX14-LIKE*	Glycosyl transferases mediating glucuronoxylan biosynthesis	Mutation analyses	Improved glucuronoxylan biosynthesis and drought tolerance in Arabidopsis.	(Keppler and Showalter, 2010)
Tobacco	*CrPOD1*	Involved in cell wall metabolism, stress response, etc.	Overexpression	Enhanced seed germination, and salinity and drought stress tolerances.	(Kumar et al., 2012).
Arabidopsis	*FERONIA (FER)*	Cell wall integrity maintenance	Mutants analysis	Maintained cell-wall integrity in *FER*-harbouring plants, whereas *fer* and *mur1* mutants showed increased root cell-wall softening and disrupted pectin cross-linking under salinity. Treatment with Ca^2+^ and borate rescued cell-wall integrity defects, and facilitated pectin cross-linking and Ca^2+^ signaling in *fer* mutants.	(Feng et al., 2018)
Arabidopsis	*HAESA-LIKE3* (*HSL3*)	A leucine-rich repeat (LRR) RLK protein	Overexpression and mutant analysis experiments.	*HSL3* negatively regulated stomatal closure and drought stress response by modulating the level of H2O2 in the guard cells.	(Liu et al., 2020)
Arabidopsis	*RLP44*	A receptor-like protein which mediates the activation of BR signaling via direct interaction with a BR coreceptor BAK1.	Mutations and GFP fusion OE analyses, coupled with confocal laser-scanning analysis.	*RLP44* activated the BR signaling module in response to PMEI overexpression-induced cell wall (pectin) perturbation.	(Wolf et al., 2014)
Arabidopsis	*AtWAKL10*	Wall-associated kinases	Knockdown mutation	The *atwakl10* knockout mutant showed enhanced tolerance to drought but reduced tolerance to salinity.	(Bot et al., 2019)
Arabidopsis	*AtRPS2*	Nucleotide-binding and leucine-rich repeat kinase	Constitutive expression	*AtRPS2*-OE transgenic plants showed higher survival rates and improved drought and salinity stress tolerance than Wt.	(Wu et al., 2023)
Wheat (*Triticum aestivum*)	*TaPOD04*	Cell wall extension, at the root apex	RT-qPCR	Sustained root growth and RWC of root tissues, and increased transcript abundance of *TaPOD04* in root apex in response to PEG-induced osmotic stress.	(Csiszár et al., 2012)
Safflower and Arabidopsis	*CtCYP71A1*	Cytochrome P450 family gene involved in biosynthesis and catabolism of secondary metabolites	Overexpression	Increased root lignin accumulation, enhanced regulation of several lignin-biosynthesis-related genes, and drought tolerance in OE safflower and Arabidopsis lines than Wt and antisense plants.	(Zhang Q. et al., 2023).
Rice	*OsNAC17*	Rice TF	Overexpression	Positively influenced several lignin biosynthesis-related genes, enhanced leaf and root lignification, and improved drought tolerance in transgenic rice.	(Jung et al., 2022)
*Paeonia ostia*	*PoWRKY17*	Transcriptional regulation of lignin and other secondary metabolism-related genes	Overexpression	Enhanced lignin accumulation, activated expression *PoCCoAOMT* genes, and drought tolerance.	(Luan et al., 2023)
Rice	*SiMYB56*	Transcriptional regulation of lignin biosynthesis related genes	Overexpression	Enhanced lignin content, ABA synthesis, expression of lignin and ABA synthesis-related genes, and drought tolerance in transgenic rice plants.	(Xu et al., 2020)
Rice	*OsTF1L*	A homeodomain leucine zipper TF involved in regulation of lignin biosynthesis	Overexpression	Elevated shoot lignin accumulation, stomatal closure, and enhanced drought tolerance in transgenic rice.	(Bang et al., 2019)
Rice	*OsERF71*	A drought-responsive AP2/ERF TF involved in regulation of lignin biosynthesis	Root-specific overexpression	Orchestrated root architecture adjustment, enhanced the expression of CW loosening and lignin biosynthetic genes, eg. *OsCCR1.*	(Lee et al., 2016)
Rice	*OsNAC10*	Rice TF	Overexpression under the control of constitutive promoter GOS2 and root-specific promoter RCc3	Root-specific overexpression enlarged roots, and enhanced rice drought tolerance and grain yield under field drought conditions.	(Jeong et al., 2010)
Rice	*OsNAC9*	Rice TF	Overexpression	Promoted root architecture adjustment and improved drought tolerance and grain yield in rice under field drought conditions.	(Redillas et al., 2012)
Rice	*OsNAC5*	Rice TF	Overexpression	*OsNAC5* activated *OsCCR10*, modulated lignin accumulation, minimized water loss rate, enhanced photosynthetic performance and vegetative stage drought tolerance in overexpressing rice plants.	(Bang et al., 2022)
Arabidopsis	*AtMYB41*	Encodes an R2R3-MYB TF involved in regulating cell expansion and cuticle deposition in response to abiotic stress	Overexpression	Transgenic OE lines showed a pleiotropic phenotype similar to that shown by mutants that affect cuticle biosynthesis, viz., enhanced sensitivity to desiccation and enhanced permeability of leaf surfaces. Expression of cuticle metabolism-, CW modification-, cell expansion-, and lipid metabolism-related genes was differentially modulated.	(Cominelli et al., 2008)
Arabidopsis	*PtoPME35*	Modulates stomatal function	Overexpression	Enhanced stomatal functioning and drought tolerance in transgenic Arabidopsis.	(Yang W. et al., 2020)
Arabidopsis	*CaPMEI1*	Pectin methyltransferase	Overexpression	Transgenic plants overexpressing *CaPMEI1* exhibited improved drought tolerance, through increased germination rate and seedling growth than Wt.	(An et al., 2008; Wormit and Usadel, 2018)
Arabidopsis	*CaXTH3*	Encodes an XTH homolog	Constitutive expression	Enhanced drought and salinity tolerance in transgenic Arabidopsis.	(Cho et al., 2006)

lew-2-1, and 2-2, leaf wilting 2-1 and leaf wilting 2-2; AnEXPA1 and AnEXPA2, Ammopiptanthus nanus expansins 1 and 2, respectively; AtWAKL10, Arabidopsis thaliana wall-associated kinase Like10; CaXTH3, Capsicum annuum xyloglucan endotransglucosylase/hydrolase 3; CLD1/SRL1, CURLED LEAF AND DWARF 1/SEMI-ROLLED; CrPOD1, Catharanthus roseus peroxidase 1; IRX14 and IRX14L, closely related glycosyl transferases in the glycosyl transferase 43 (GT43) family of Arabidopsis; RPK1, receptor-like protein kinase 1; RLP44, receptor –like protein 44; leaf RWC, leaf relative water content; OE, overexpressing; OsCCR1, OsCINNAMOYL-COENZYME A REDUCTASE 1; OsTF1L, Oryza sativa transcription factor 1-like; OsERF71, Oryza sativa drought-responsive AP2/ERF transcription factor; PtoPME35, Populus tomentosa PME35; P5CS, pyrroline-5-carboxylate synthase; SDR1, alcohol dehydrogenase/reductase; SiMYB56, Setaria italic MYB56; RT-qPCR, real-time quantitative PCR. Table modified from (Houston et al., 2016; Wang et al., 2016).

## 4 Cell wall modifications necessary for plant drought tolerance

Drought stress response-associated cell wall plasticity conceivably contributes to cell turgor maintenance (Martínez et al., 2007), and as such, can be connected with plant drought tolerance (Le Gall et al., 2015). In common beans (*Phaseolus vulgaris* L.), for example, a comparative analysis of six genotypes showed that more drought tolerant genotypes had a huge drop in elasticity modulus (**ϵ**), coupled with greater cell wall elasticity, which enabled them to maintain better their turgescence (Martínez et al., 2007). In comparison, drought susceptible genotypes did not display any significant decrease in ϵ or cell wall elasticity, suggesting that cell wall elasticity may be critical for cell integrity maintenance and drought stress tolerance (Martínez et al., 2007; Hessini et al., 2009; Le Gall et al., 2015). Meanwhile, several enzymes, proteins and ions such as PMEs, expansins, β-glucanases, PODs, Ca^2+^ ions, etc. regulate cell wall remodeling-related loosening and stiffening processes that are critical for inducing growth changes, and drought tolerance (Tenhaken, 2014; Wu et al., 2018; Ganie and Ahammed, 2021; Qiu et al., 2021). Here, various forms of these cell wall modifications are discussed.

### 4.1 Cell wall pectin ester modifications

Cell wall pectin ester modifications are mainly achieved through the processes of demethylesterification and *O*-acetylation, discussed hereunder this section.

#### 4.1.1 Demethylesterification by pectin methylesterases

A stress-challenged cell institutes specific cell-wall-protein-biosynthesis-related transcriptional responses, which modify cell wall components, including pectin, and significantly alter the cell wall architecture (Le Gall et al., 2015; Novaković et al., 2018; Wu et al., 2018; Baez et al., 2022; Wu et al., 2022). Pectin modifications are mediated by a large family of cell-wall-localized enzymes, the PMEs, which catalyze the removal of methyl esters from the d-GalA backbone of HG (Braidwood et al., 2014), with their activity being controlled by pectin methylesterase inhibitors (PMEIs) (Sénéchal et al., 2015; Wormit and Usadel, 2018). PMEs regulate apoplastic Ca^2+^ levels in response to stress (Wu and Jinn, 2010; Wu et al., 2018). The action of PMEs yields carbanions on the HG, permitting the creation of Ca^2+^ cross-linking networks of unmethylesterified GalA units (Braidwood et al., 2014; Wormit and Usadel, 2018; Cao L. et al., 2020; Kumar et al., 2023), which increases cell wall stiffness (Braybrook and Peaucelle, 2013). This possibly increases cell wall water preservation and limits dehydration. On the other hand, pectin breakdown, due to PME hydrolysis of pectic HG, leads to cell wall loosening and extensibility (Ezaki et al., 2005; Pelloux et al., 2007; Peaucelle et al., 2012). This pectin-induced alteration in cell wall mechanics essentially modulate cell wall growth and response to desiccation (Wu et al., 2018; Liu X. et al., 2021). However, to what extent this pectin-modification-induced cell wall flexibility under water deficit conditions effect plant drought tolerance is yet to be clarified.

Higher levels of demethylesterified HG were observed upon desiccation in desiccation-tolerant plant *Craterostigma plantagineum*, but this was reversed after rehydration (Jung et al., 2019). A greater amount of de-methylesterified HG, upon desiccation, and when combined with Ca^2+^ ions, results in the creation of pectate gel-like structures known as “egg-boxes”, which essentially regulate wall biomechanics and cell-cell bonding to enhance cell wall stiffness (Moore et al., 2008; Jung et al., 2019; Chen et al., 2020; Du et al., 2020), which may be critical in preserving cell water, minimizing dehydration and enhancing drought tolerance. Highly de-methylesterified HG also offers extra binding sites for pectin binding proteins (Pelloux et al., 2007), which may be crucial in perceiving cell wall hydration status (Chen et al., 2020). PME-mediated cell-wall demethylesterification has been shown to crucially regulate abiotic stress tolerance, including heat (Huang et al., 2017; Wu et al., 2018), stem lodging (Hongo et al., 2012), salinity (Yan et al., 2018b), etc. Besides, PMEs are also frequently modified in response to drought stress (Tenhaken, 2014; Ezquer et al., 2020). For instance, *Capsicum annuum* (pepper) gene *CaPMEI1* is transcriptionally induced by drought stress, and may be involved in plant drought tolerance (An et al., 2008). Compared to Wt control, *CaPMEI1*-OE Arabidopsis lines displayed improved drought tolerance, evidenced by increased germination rate and seedling root growth (An et al., 2008). In poplar (*Populus tomentosa)* and Arabidopsis, *PtoPME35* modulates stomatal functioning and drought response (Yang W. et al., 2020). Pectin methylesterification degree is reduced in *PtoPME35*-OE transgenic poplar plants, whereas overexpressing *PtoPME35* in Arabidopsis inhibits stomatal opening, resulting in reduced leaf transpiration under drought stress conditions (Yang W. et al., 2020). In tomato (*Solanum lycopersicum* L.), transient silencing of the PMEI gene *Slpmei27* by virus-induced gene silencing significantly improves drought resistance, through altering cell wall structure, stomatal permeability, and ROS balance, as well as reducing water loss rate (Cheng et al., 2022).

*PME35*-mediated PCW demethylesterification also regulates mechanical ability of Arabidopsis stems to resist lodging (Hongo et al., 2012), whereas *PMEI5*-mediated pectin modification enhances dehydration tolerance in onion (Forand et al., 2022), suggesting that it could also play a role in improving drought tolerance. It is known that increased aggregates of water bound within the cell wall preserves tissue hydration and turgor pressure, thereby enhancing cell wall rigidity (Ortega, 2010; Thompson and Islam, 2021). It has been shown that reduced cell-wall pectin content inhibits stress-induced root cell growth (Liu X. et al., 2021). Compared to Wt or mutants with decreased cellulose content, Arabidopsis mutants with decreased pectin or hemicellulose content exhibited no root cell growth under drought conditions suggesting that an appreciable quantity of pectin is needed for root cell growth under drought stress conditions (Liu X. et al., 2021). Although no relationship evidently linked the levels of pectin methylesterification to cell growth, analysis of cell wall composition, coupled with 2β-deoxy-Kdo experiments, suggested that RGII could play a crucial role in these processes (Liu X. et al., 2021). Remarkably, a comparative study in wheat revealed that, compared to drought-sensitive variety Creso, the percentages of RGI and RGII side chains were considerably increased in the drought-resistant variety Capeiti in response to water stress, supporting the role of pectin side chains in drought stress response (Leucci et al., 2008), which is possibly creation of hydrated gels that minimize cell damage (Leucci et al., 2008; Caffall and Mohnen, 2009; Tenhaken, 2014).

#### 4.1.2 Pectin *O*-acetylation by pectin acetylesterases

Besides the methylesterification and demethylesterification of the α-(1,4)-linked GalA at the *O*-6 sites of the backbone, which influence the formation of Ca^2+^ bridges between HGs (Miao et al., 2011), occasional acetylation of pectin at *O*-2 and *O*-3 sites also occur as an essential architectural and functional feature of pectin (Zhang et al., 2021b; Shahin et al., 2023). *O*-acetylation of pectin also leads to the formation of pectic gel, and is mediated by the pectin acetylesterases (PAEs), as well as the Trichome-Birefringence Like (TBL) and Reduced Wall Acetylation (RWA) family proteins (Bischoff et al., 2010; Stranne et al., 2018). For instance, investigations of *tbl* mutants in Arabidopsis have shown that plant dwarfism, weak stems, and stunted growth are linked to the deficiency of TBL genes (Bischoff et al., 2010). This implies that pectin *O*-acetylation considerably impacts plant morphogenesis, development, and responses to abiotic and biotic stresses (Gille and Pauly, 2012; Shahin et al., 2023). In Arabidopsis, *TBL10* (*AT3G06080*) has been shown to orchestrate *O*-acetylation of RGI and abiotic stress responses (Stranne et al., 2018). Compared to Wt plants, *tbl10* mutants, displaying reduced RGI *O*-acetylation, had increased drought tolerance levels, suggesting that this alteration (*O*-acetylated RGI) may impact water uptake and transport (Stranne et al., 2018).

Plant cell wall O‐acetylation is also adjusted in response to drought stress. For instance, *O*‐acetylation level in *Populus trichocarpa* leaf cell walls was significantly elevated in response to drought stress (Jardine et al., 2022). This rapid response to stress, similar to other mechanisms of cell wall methylation of polysaccharides, could provide the plasticity necessary for plant growth changes and stress adaptation, including stomatal closure (Jardine et al., 2022), and influencing photosynthesis and water relations (Roig-Oliver et al., 2020; Ganie and Ahammed, 2021). Besides, plant cell wall *O*-acetylation is known to crucially regulate physicochemical, mechanical and architectural processes essential for curtailing degradation, simultaneously promoting intermolecular crosstalk among cell-wall polymers (Biely, 2012; Peaucelle et al., 2012; Shahin et al., 2023). Meanwhile, Arabidopsis *PAE2*, *PAE4*, and *PAE8* have been shown to be induced, and highly expressed, by osmotic stress (Philippe et al., 2017). Considering that these PAEs may also have important roles in photosynthesis (Roig-Oliver et al., 2020), it will be plausible and interesting to functionally characterize these or other phylogenetically-related PAEs under drought stress conditions.

### 4.2 Cell wall loosening and stiffening

#### 4.2.1 Xyloglucan endotransglucosylases and expansins underpin cell wall loosening-mediated morphogenesis and stress response

Plant cell and organ morphogenesis, under both benign and stress conditions, requires a specialized cell wall remodeling mechanism that relaxes cell wall tensions created by turgor pressure, permitting water influx to reestablish cell wall tension and cell expansion (Cosgrove, 2016a; Chebli and Geitmann, 2017). Cell wall loosening is a form of wall modification that achieves this purpose and underpin creep, ie., a protein-mediated process and irrevocable time-dependent cell extension (Cosgrove, 2018; Zhang et al., 2019). The loosening of cell wall polysaccharides is suggested to play a crucial role under osmotic, drought, or salinity stresses, that is, to sustain the possibility for cells and organs to expand under those conditions (Tenhaken, 2014). This cell wall loosening is mediated by cell wall proteins XTHs, β-glucanases, xyloglucan endo-transglycosylases (XETs), EXPAs, etc., which orchestrate cell turgor-driven cell enlargement (Cosgrove, 2000; Ezquer et al., 2020; Stratilová et al., 2020), and appear to be regulated by drought stress as observed in soybean (Coutinho et al., 2021). The XTHs, β-glucanases, and XETs regulate the remodeling of primary load-bearing components pectin matrix and cellulose/xylogucan network, to generate the morphological alterations necessary for plant development and stress defence (Chebli and Geitmann, 2017). By cleaving and ligating non-load-bearing xyloglucans, these proteins characteristically reduce the number of linkages between cellulose and the load bearing components, resulting in a more easily breakable wall. On the other hand, EXPAs induce creep (Cosgrove, 1999; Cosgrove, 2016b; Samalova et al., 2022).

Expansins (EXPs) modulate cell wall loosening by inducing cell wall stress relaxation and extension in a pH-dependent manner (Rayle and Cleland, 1992; McQueen-Mason and Cosgrove, 1995; Cosgrove, 2000; Cosgrove, 2015; Cosgrove, 2016a). Particularly, among the four different subgroups of plant expansins, viz., EXPA (α-expansin), EXPB (β-expansin), EXLA (expansin-like A), and EXLB (expansin-like B) (Kende et al., 2004; Sampedro and Cosgrove, 2005), EXPAs and EXLBs have been central in inducing cell wall loosening, via acid growth response or auxin-induced acidification of the cell wall space (McQueen-Mason et al., 1992; Rayle and Cleland, 1992; Cosgrove, 2016a; Chebli and Geitmann, 2017). EXPAs are the most abundant, and have been characterized in different crop species such as Arabidopsis, rice, wheat, poplar, etc. (see (Marowa et al., 2016; Samalova et al., 2022)). Meanwhile, the cell-wall loosening model (Cosgrove, 2015) enunciates that non-water-stressed quiescent cells are at osmotic balance, as wall stresses offset the externally exerted turgor pressure on the wall. However, in growing cells, the cells are loosened through the EXPs-modulated pH-dependent manner, which involves relaxation of the load-bearing cell wall components and release of turgor-generated wall stresses, allowing water flow into the cell and restoration of wall tension and cell expansion (Cosgrove, 2015; Cosgrove, 2018; Samalova et al., 2022). Although EXPs lack the capacity to hydrolize the polysaccharide substrates by themselves (McQueen-Mason et al., 1992; McQueen-Mason and Cosgrove, 1995), pH shifts facilitate EXP-mediated cell wall loosening, via cell wall components relaxation, which enables access to polysaccharide substrates by different hydrolases (Cosgrove, 2000; Cosgrove, 2005; Samalova et al., 2022).

Several EXPAs transcripts are up-regulated under abiotic stress (Tenhaken, 2014; Marowa et al., 2016), with enhanced EXPAs expression contributing to drought tolerance ( (Samalova et al., 2022) and references therein). For instance, overexpression of *TaEXPA2* promotes drought tolerance in *TaEXPA2-*OE wheat plants, by improving cell water retention, antioxidant capacity, and lateral root proliferation under drought stress (Yang J. et al., 2020). When overexpressed in tobacco, *TaEXPA2* enhances water deficit tolerance and seed production, by promoting osmotic adjustment, antioxidant capacity, and expression of numerous antioxidant-enzymes-encoding genes (Chen et al., 2016). *TaEXPB23* overexpressed in tobacco enhanced water deficit tolerance, by reducing the rate of water loss (Li et al., 2011). Overexpression of *GhEXLB2* improved drought tolerance in cotton (*Gossypium hirsitum* L.), by enhancing WUE, soluble sugar and chlorophyll contents (Zhang et al., 2021a). An *Erianthus arundinaceus* EXPA gene (*EaEXPA1*) overexpressed in sugarcane (*Saccharum* spp. cv. Co 86032) enhanced drought tolerance in transgenic sugarcane lines, via improved leaf relative water content and photosynthetic parameters (Ashwin Narayan et al., 2021). *Ammopiptanthus nanus* EXPA genes *AnEXPA1* and *AnEXPA2* overexpressed in Arabidopsis enhanced cold and drought tolerance in transgenic Arabidopsis plants, with *AnEXPA2* being induced by both cold and drought, and responding to hormone induction (Liu et al., 2019); *AnEXPA2* was suggested to enhance drought tolerance by improving ROS scavenging ability and EXP activity in overexpressing transgenic plants (Liu et al., 2019). Equally, an EXP gene *AstEXPA1* from creeping bentgrass (*Agrostis stolonifera*), overexpressed in tobacco, improved tolerance to drought (and other stresses) by increasing soluble sugar content and osmoprotection in transgenic plants (Hao et al., 2017). What is revealing from these few examples is that progenitors may harbor important cell-wall loosening-related genes that can be harnessed for improving drought tolerance in elite crop species (Tucker et al., 2018).

Meanwhile, extensins (eg. leucine-rich repeat extensins, LRXs) are crucial hydroxyproline-rich glycoproteins that are known to reinforce plant cell walls, by forming intra and intermolecular crosslinking of tyrosine residues (Lamport et al., 2011; Castilleux et al., 2021), thereby improving mechanical protection against pathogen attack (Castilleux et al., 2021). Besides, LRXs interact with RALF peptide ligands that modify cell wall expansion (Moussu et al., 2023), and synergistically link with transmembrane receptor FER in cell growth regulation (Moussu et al., 2023). This may suggest that LRXs coordinate the cell-wall-PM connection that underpins extracellular signal perception or information transfer necessary to steer cell expansion or/and cell wall formation (Draeger et al., 2015; Herger et al., 2019). However, it remains to be examined whether extensins can steer cell growth under drought stress conditions. Nonetheless, the most plausible role of extensins in drought tolerance, due to their capacity to form cross-linked dendritic assemblies with peroxidases (Mishler-Elmore et al., 2021), may be cell wall stiffening, which enhances cell water preservation. Overall, several cell-wall-related proteins crucially regulate cell wall extensibility, by mediating cell turgor maintenance, enlargement and expansion, as well as morphological and physiological alterations necessary for both growth and stress response (Martínez et al., 2007; Le Gall et al., 2015; Chebli and Geitmann, 2017; Ezquer et al., 2020), and these could be targeted for genetic or metabolic modulation to improve plant drought stress tolerance.

#### 4.2.2 The role of peroxidases and laccases

Cell wall PODs, ROS, and laccases (LACs) are also involved in cell wall loosening and stiffening (Tenhaken, 2014; Francoz et al., 2015; Xie et al., 2018). Due to their twofold (hydroxylic and peroxidative) catalytic cycles (Passardi et al., 2004), PODs may generate oxidative radicals (OH^•^, ·O2^–^, etc.), and at the same time oxidize glycoproteins or phenolics esterified with cell wall aromatic compounds (monolignols, cinnamic acids, aromatic amino acids, etc.) that are free or polysaccharides-linked (Tenhaken, 2014; Francoz et al., 2015). Thus, conceptually, it has been enunciated that under stress conditions, plant cellular growth is tightly coordinated by the antagonism or delicate balance between these ROS or POD-mediated cell wall stiffening and weakening processes (Tenhaken, 2014). On one hand, crosslinking of glycoproteins to polysaccharides-esterified phenolic compounds relies on LACs- or POD-generated ROS (oxygen radicals, ·OH) (Vanholme et al., 2010), yielding to cell wall rigidification, whereupon expansins or XTHs access to xyloglucan substrate is hindered, and cell growth is arrested (Tenhaken, 2014; Francoz et al., 2015; Alavarse et al., 2022). On the other hand, prolonged stress and ROS synthesis cause POD substrates depletion, which favor hydroxyl radicals formation (H_2_O_2_-driven); and the formed hydroxyl radicals induce direct cleavage of cell wall polysaccharides, through covalent bonds breakage, resulting in cell wall loosening, and consequent cell expansion almost similar to non-stress conditions (Schopfer, 2001; Tenhaken, 2014; Samalova et al., 2022). Thus, PODs crucially modulate cellular H_2_O_2_ and ROS homeostasis under stress conditions (Kidwai et al., 2020).

LACs, in concert with PODs, crucially catalyze monolignol polymerization in the apoplastic cell wall domains, to facilitate cell wall lignification (Wang et al., 2013; Zhao et al., 2013; Xie et al., 2018). In this process, monolignols are actuated by LAC or POD oxidation systems to produce the final lignin polymers (Tobimatsu and Schuetz, 2019). The ensuing cell wall stiffening or rigidification helps plants to resist drought stress, possibly by waterproofing tissues (Le Gall et al., 2015). Evidence from multiple LACs or PODs mutants-based genetic studies show that LACs and PODs cooperate in cell wall lignification. For instance, *LAC4* and *LAC17* crucially regulate tissue-specific lignin accumulation in Arabidopsis (Berthet et al., 2011). Double knockout (*LAC4* and *LAC17*) mutants had ~20-40% reduction in stem lignin content when compared to Wt (Berthet et al., 2011). In concert with *LAC11*, they exhibit high expression in lignifying tissues, and, simultaneous disruption of *LAC11*, *LAC4* and *LAC17* severely arrests plant growth and vascular development, and considerably diminish root lignin accumulation (Zhao et al., 2013). Intriguingly, putative lignin POD genes were expressed at normal or higher levels in the LAC triple mutant, signifying that lignin LAC activity is essential and non-redundant with POD activity for stem and root vascular tissue lignification in Arabidopsis (Zhao et al., 2013). Similarly, quadruple and quintuple loss-of-function Arabidopsis mutants revealed that *LAC5*, *LAC10*, and *LAC12* non-redundantly modify lignin accumulation in distinct lignified cell types (Blaschek et al., 2023). Meanwhile, Arabidopsis POD genes *AtPOD2*, *AtPOD25* and *AtPOD71* cooperatively modulate stem lignification (Shigeto et al., 2015), with three double mutants (*atPOD2/atPOD25*, *atPOD2/atPOD71*, and *atPOD25/atPOD71*) resulting in ~ 11-25% decrease in stem lignin content (Shigeto et al., 2015). Besides, *AtPOD17* essentially mediates leaf, stem, flower, and silique lignification (Cosio et al., 2017), whereas *AtPOD64* regulates Casparian strips lignification (Lee et al., 2013). All these observations suggest that different LAC and POD genes play prominent lignification-related roles in distinct cell- or tissue-types (Xie et al., 2018; Blaschek et al., 2023; Choi et al., 2023).

Meanwhile, overexpression of POD genes has been shown to enhance crop drought tolerance. For instance, several POD genes were up-regulated under heat, salinity and drought stresses in potato (*Solanum tuberosum*); specifically, five genes (*StPRX19*, *StPRX28*, *StPRX40*, *StPRX41*, and *StPRX57*) were upregulated under drought conditions (Yang X. et al., 2020), suggesting they could play a role in drought tolerance. However, their exact regulatory functions will need to be investigated. Microarray investigation revealed five candidate genes (*ZmPRX26*, *ZmPRX42*, *ZmPRX71*, *ZmPRX75*, and *ZmPRX78*) whose expression is considerably altered in response to both 20 mM NaCl and 20% PEG treatments in maize, particularly being highly expressed in the roots, suggesting their involvement in root-related salinity and drought stress responses (Wang et al., 2015). It may be that these *ZmPRX* genes regulate maize drought tolerance by enhancing root lignin accumulation, similar to *AtPOD64* (Wu Y. et al., 2017), which promotes root system development and stress tolerance (Wu Y. et al., 2017). Arabidopsis seedlings with knocked out *AtPOD33* exhibited shorter roots than WT controls, whilst seedlings overexpressing *AtPOD34* exhibited significantly longer roots (Passardi et al., 2006), with the root length modifications linked to corresponding cell length changes (Passardi et al., 2006). Overexpression of the *AtPOD64* gene also increased root growth in Arabidopsis (Wu Y. et al., 2017). It is well known that a well-developed root system can improve plant drought tolerance (Wasaya et al., 2018). Conceivably, overexpressed *PODs* enhance root elongation, which improves deeper water extraction, whereas root lignification helps preserve axial water transport, thereby improving crop drought tolerance (Zhao, 2016).

### 4.3 Stomata guard cell wall remodeling

Plant cell walls play a very important role in stomatal opening, a key process that determines drought resistance (Alonso Baez and Bacete, 2023). Stomata, which are small orifices localized on the leaf epidermis of plants, and bounded by two guard cells, facilitate an interface for plant-environmental gas exchange, thereby governing plant water balance (Auler et al., 2022; Liu C. et al., 2023). Dynamic alteration of guard cell wall (GCW) structure controls stomatal conductance, photosynthesis, and water loss frequencies, as well as pathogen entry (see details in (Jaafar and Anderson, 2024)). The opening and closing of stomata orifices is mediated by changes in turgor pressure, structure and composition of the two guard cells, as affected by various signals such as ABA, ROS, Ca^2+^, blue light and extracellular calmodulin (Kollist et al., 2014; Liu et al., 2022). Since there is generally reduced turgor pressure under drought stress conditions, stomatal closure is amplified (as the first reaction to drought stress) to minimize water loss from transpirational pathways and maintain turgor (Pirasteh-Anosheh et al., 2016). This improves water use efficiency and drought tolerance (Auler et al., 2022). GCWs are dynamically remodeled to facilitate this process. For instance, the differential thickening and orientation of cellulose microfibrils gets ramped up, permitting guard cells to act reversibly during repeated stomatal opening and closing (Jaafar and Anderson, 2024). Especially, the anisotropic behavior of GCWs (being elastic to structural and orientational modulation of cellulose microfibrils (Baskin, 2005)) allows for this stomatal functioning, through continuous swelling and deflation during drought stress episodes (Lawson and Matthews, 2020).

**Figure 3:**
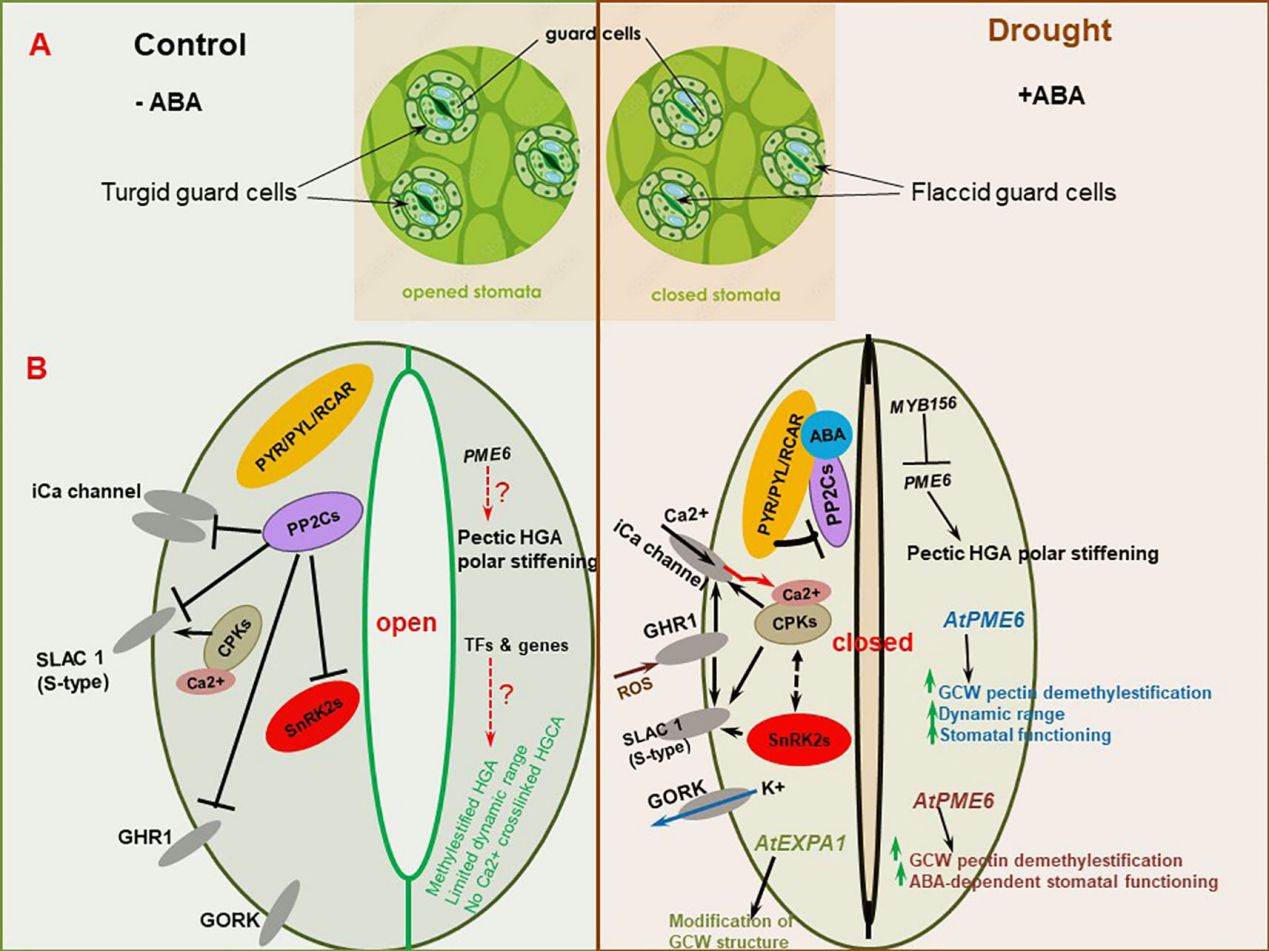
Guard cell wall (GCW) remodeling-mediated stomatal regulation. **(A)** Illustration of guard cell structure under control and drought stress conditions. **(B)** Abscisic acid (ABA) and GCW remodeling-mediated mechanisms regulating stomatal functioning under stress conditions. The canonical PYR/PYL/RCAR-mediated ABA signaling module regulates stomatal regulation in alliance with reactive oxygen species (ROS), calcium (Ca^2+^) signaling and GCW remodeling processes. Modulation of guard cell pectic matrix results in altered GCW properties, which consequently influence mechanical stomatal responses to exogenous cues, thereby regulating stomatal functioning. For simplicity/clarity purposes, the ABA-mediated signaling pathway is shown on the left side whereas the CW remodeling mechanisms are shown on the right side of each guard cell, respectively; however, in reality, these pathways are crosslinked and are distributed across entire guard cell. Dashed red arrow and question mark imply that the signaling pathway needs further clarification. Green upward-pointing arrows denote increased or improved trait/physiological process. The red arrow in Figure B (bottom right) represent cytosolic free calcium [Ca^2+^]_cyt_ outburst. PYR/PYL/RCAR, pyrabactin resistance/pyrabactin resistance-like/regulatory component of ABA receptors; PP2Cs, type 2C protein phosphatases; SnRK2s; sucrose non-fermenting 1 (SNF1)-related protein kinases 2; CPKs, calcium protein kinases; GHR1, GUARD CELL HYDROGEN PEROXIDE-RESISTANT 1 channel; GORK, GUARD CELL OUTWARD RECTIFYING K+ CHANNEL; SLAC1 (S-type), slow anion channel 1 (slow type); iCa channel, Ca^2+^-permeable cation (iCa) channel; *AtEXPA1*, *Arabidopsis thaliana* expansin A1; *AtPME6*, *Arabidopsis thaliana* pectin methyltransferase 6; HG, homogalacturonan. Illustrations are based on (Cotelle and Leonhardt, 2019) (for ABA signaling), and (Amsbury et al., 2016; Carter et al., 2017; Zheng et al., 2023) (for pectin modifications).

Meanwhile, Arabidopsis GCWs are endowed with unesterified HGs and arabinans, whilst methyl-esterified HGs and Ca^2+^ cross-linked blockwise de-esterified HGs are restricted to the exterior of the wall (Amsbury et al., 2016; Merced and Renzaglia, 2019). Under stress conditions, guard cells undergo recurring inflation-deflation cycles, with this process and the formation of guard cell orifice being necessitated by the modification of pectic HG. Augmenting pectic HG modification, via demethyleesterification, promotes pore formation, whereas hindering HG demethylesterification defers pore initiation and impedes pore enlargement (Rui et al., 2019). This may suggest that stomatal function is governed by GCW characteristics, and involves GCW demethylesterification (Amsbury et al., 2016; Lawson and Matthews, 2020) (**Figure 3**). GCW-expressed *AtPME6* is necessary for stomata opening and closing. GCWs of *pme6-1* mutant are enriched with methylesterified pectin and exhibit a decreased dynamic range when exposed to stomata closing/opening elicitors, suggesting the nullification of stomatal function to be a result of a GCW mechanical alteration (Amsbury et al., 2016). Similarly, a GCW-specific PME, *AtPME53*, crucially drives ABA-dependent stomatal function and heat stress response in Arabidopsis (Wu et al., 2022). This reveals a link between PME-mediated GCW pectin demethylesterification, stomatal functioning, and abiotic stress response (Amsbury et al., 2016; Wu et al., 2022). Besides, *PME34* crucially regulates GCW plasticity modulating stomatal conductance in response to abiotic stress such as heat in Arabidopsis (Huang et al., 2017; Wu H-C. et al., 2017). Equally, a GCW-expressed *AtEXPA1* controls stomatal functioning in Arabidopsis through modifying GCW structure (Wei et al., 2011). These studies show that PME and EXPAs activities regulating GCW remodeling are essential stress response strategies (Wu et al., 2022), and hence, could be possible targets for deliberate genetic manipulation to engineer drought tolerance in crops.

More recently, it has been shown that *MYB156* TF controls pectic HG-based polar stiffening in poplar (*Poplar* sp*ecies*), via downregulation of *PME6* (Zheng et al., 2023). Polar stiffening is necessary for guard cell dynamics critical for normal stomatal morphology maintenance during stomatal movement in response to alterations in environmental conditions (Carter et al., 2017). *MYB156*-deficient plants had increased stomata polar stiffness and improved stomatal dynamics and response rate to environmental cues, whereas *MYB156-*overexpressed plants had decreased stomata polar stiffness and lessened stomatal dynamics, revealing the connection between the GCW structure and function in stomatal dynamics (Zheng et al., 2023) (**Figure 3**). This study provides a potential way for enhancing plant stomatal functioning and water deficit tolerance by engineering this specific property (Zheng et al., 2023). Taken together, the GCW properties underpin the dynamic stomata guard cell deformations that occur during guard cell expansion and contraction to drive stomatal opening and closing, whose precise regulation is critical for water transport, photosynthesis and growth, especially under drought conditions (Siqueira et al., 2021; Jaafar and Anderson, 2024), and could be appropriate targets for manipulation through genetic engineering approaches. It will be crucial to establish the crosstalk existing between GCW remodeling mechanisms, ABA signaling and CWI sensing system as it sheds more light on stomatal functioning dynamics and drought response.

### 4.4 Lignification

**Figure 4:**
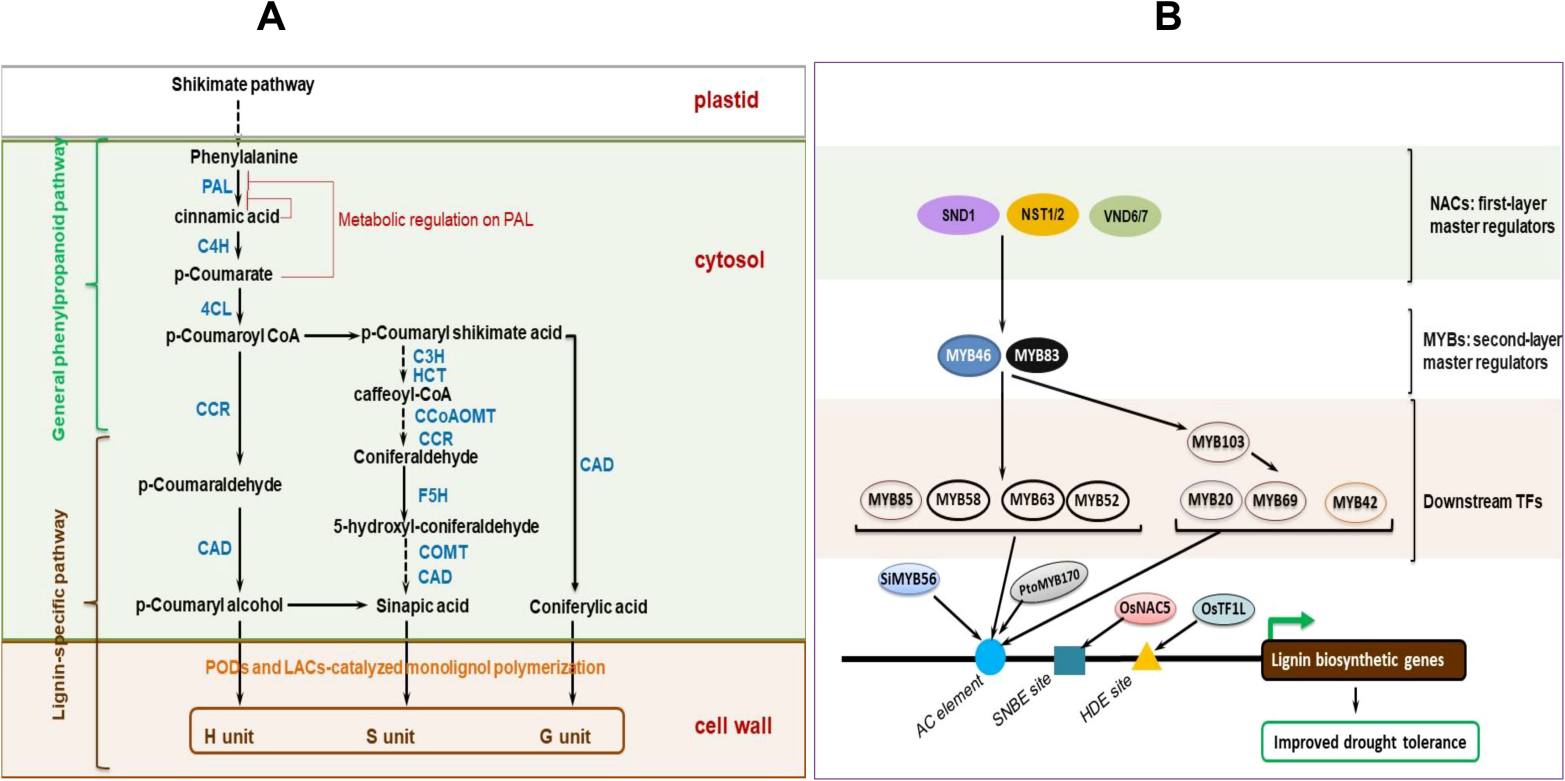
Lignin biosynthesis and modulation-mediated drought tolerance in plants. **(A)**. Simplified pathway for lignin biosynthesis in plants. Phenylalanine ammonia lyase (PAL) act on the phenylalanine generated from the shikimate pathway to produce cinnamic acid. Downstream, diverse enzyme superfamilies catalyze series of steps to convert cinnamic acid into monolignols (p-Coumaryl alcohol, sinapic acid, and coniferylic acid), which are subsequently polymerized (through class III peroxidases- and laccases-mediated oxidative radical coupling) into lignin units (p-hydroxyphenyl, H; guaiacyl, G; and syringyl, S). The resultant lignification stiffens the cell wall. Note: Key enzymes (not exhaustive) are displayed in blue text, as follows: PAL, phenylalanine ammonia lyase; C4H, cinnamate 4-hydroxylase; 4CL, 4-coumaroyl CoA-Ligase; CCR, cinnamoyl CoA reductase; CAD, (hydroxy)cinnamyl alcohol dehydrogenase; C3H, p-coumaroyl shikimate/quinate 3-hydroxylase; HCT, hydroxycinnamoyl-CoA shikimate/quinate hydroxycinnamoyl transferase; CCoAOMT, caffeoyl CoA O-methyltransferase; CCR, cinnamoyl-CoA reductase; F5H, ferulate 5-hydroxylase; COMT, caffeic acid/5-hydroxyferulic acid O-methyltransferase. Complete arrows signify single-step enzymatic reactions, whereas dashed arrows imply series of enzymatic steps (not fully shown) for clarity purposes. Figure adopted from (Nguyen et al., 2016; El-Azaz et al., 2020) and others discussed in text. **(B)** NAC-MYB-mediated transcriptional regulation of lignin biosynthetic genes involved in plant drought tolerance. Transcription factors bind to the cis-acting elements (TF binding sites), viz., AC element, secondary wall NAC binding element (SNBE), and HD-binding cis-elements (HDE) on the promoter of lignin biosynthetic genes to actuate their expression. TFs without prefixes relate to Arabidopsis (*Arabidopsis thaliana*) whereas those with prefixes are for other species. *SiMYB56*, *Setaria italic MYB56*; *OsNAC5*, *Oryza sativa NAC5*; *PtoMYB170*, *Populus tomentosa MYB170; OsTF1L*, *Oryza sativa transcription factor 1-like*; SND1, SECONDARY WALL-ASSOCIATED NAC DOMAIN PROTEIN 1; NST1/2, NAC SECONDARY WALL THICKENING PROMOTING FACTOR 1/2; VND6/7, VASCULAR-RELATED NAC DOMAIN 6/7. Illustrations are based on (Nakano et al., 2015; Yoon et al., 2015; Han et al., 2022; Choi et al., 2023) and others discussed in text.

Lignin is biosynthesized through the general phenylpropanoid pathway (Vanholme et al., 2010) (**Figure 4A**). This pathway is mediated by several key enzymes, including phenylalanine ammonia lyase (PAL), ferulate 5-hydroxylase (F5H), p-coumarate 3-hydroxylase (C3H), 4-coumarate coenzyme A ligase (4CL), p-hydroxycinnamoyl-CoA:quinate/shikimate hydroxycinnamoyl transferase (HCT), cinnamate 4-hydroxylase (C4H), caffeoyl-CoA O-methyltransferase (CCoAOMT), caffeic acid O-methyltransferase (COMT), cinnamoyl-CoA reductase (CCR), cinnamyl alcohol dehydrogenase (CAD), etc (Dixon et al., 2001; Vogt, 2010; Wang et al., 2013; Dong and Lin, 2021; Han et al., 2022). Consequently, genetic modification of these enzymes has been shown to significantly alter lignin accumulation and/or composition, as well as stress defense (Xie et al., 2018). Monolignols secreted by lignifying cells are polymerized by cell-wall-localized O_2_-reliant laccases (LACs) and H_2_O_2_-dependent peroxidases (PODs) into lignin polymers (Tobimatsu and Schuetz, 2019; Han et al., 2022) (**Figure 4A**). Lignification involves the deposition of lignin into apoplastic cell wall domains (Vanholme et al., 2010), rendering them mechanically strong, firm, and hydrophobic (Zhao, 2016); this stiffens cell wall extensibility, and controls cell membrane permeability and water passage to maintain cell osmotic balance (Moura et al., 2010; Bang et al., 2022). Increased lignin accumulation helps plants adapt to drought stress (Xu et al., 2017). For instance, overexpression of poplar (*Populus* L.*)* transcription factor *PdNF-YB21* promoted root lignification, enlarged xylem vessels and root growth, which improved drought tolerance (Zhou et al., 2020). Additionally, *PdNF-YB21-PdFUS3-PdNCED3* module promoted root ABA content, auxin regulation of root growth and drought tolerance in poplar (Zhou et al., 2020). A *Setaria italic* transcription factor, *SiMYB56*, when overexpressed in rice, confers drought tolerance, by lowering MDA content, increasing lignin content, and modulating lignin biosynthesis and ABA signaling pathway under drought conditions (Xu et al., 2020).

NAC and MYB TFs crucially modulate lignin biosynthesis, essential for drought stress tolerance in plants (**Figure 4B**). For instance, *OsNAC5* TF actuates a rice gene *OsCCR10* (*Cinnamoyl-CoA Reductase 10*) and enhances vegetative stage drought tolerance in overexpressing rice plants, by modulating root lignin accumulation (via increased H- and G-lignin biosynthesis), greater photosynthetic performance, and minimized leaf water loss rate than in non-transgenic controls (Bang et al., 2022). *O*verexpression of *OsNAC17* positively influences numerous lignin biosynthesis-related genes, enhances leaf and root lignification, and improves drought tolerance in rice (Jung et al., 2022). Besides, ectopic expression of an HD-Zip TF *OsTF1L* elevates lignin biosynthesis, lignin accumulation in shoots, and stomatal closure, which enhances rice reproductive-stage drought tolerance (Bang et al., 2019). Equally, an overexpressed cytochrome P450 family gene *CtCYP71A1* results in increased root lignification, positive regulation of several other lignin biosynthetic genes, and improved drought tolerance in overexpressing safflower (*Carthamus tinctorius*) and Arabidopsis lines than in wild-type and antisense plants (Zhang Q. et al., 2023). Overexpressing *Paeonia ostia* (an oil-producing woody crop) gene *PoWRKY17* activates *PoCCoAOMT* expression, enhances lignin accumulation, increases leaf RWC, decreases relative electrical conductivity, minimizes MDA content and ROS accumulation, but enhances protective enzyme activities. All these positive physiological changes endow the crop plants with improved water deficit tolerance (Luan et al., 2023). Besides, a glycine-rich RNA-binding protein, *OsGRP3*, promotes rice drought tolerance by modulating phenylpropanoid biosynthesis and enhancing lignin accumulation (Xu W. et al., 2022). Upregulated expression of phenylpropanoid biosynthesis-related genes (*PAL1*, *4CL, CCR, C4H*, *CCOMT*, and *CAD6*) improved lignin biosynthesis and confered cluster bean [*Cyamopsis tetragonoloba* (L.) Taub.] drought tolerance, by increasing water-flow resistance and maintaining steady nutrient transport under drought stress conditions (Narayan et al., 2023). In apple (*Malus* × *domestica* Borkh.), *MdMYB88* and *MdMYB124* improve water deficit tolerance by regulating root xylem development and root cell wall cellulose and lignin depositions (Geng et al., 2018). Overexpressing a CCCH-type TF, *PuC3H35*, promotes root proanthocyanidin (an effective non-enzymatic antioxidant) and lignin biosynthesis, as well as vascular tissue development, which confers improved drought stress tolerance in *Populus ussuriensis*, by actuating anti-oxidation and mechanical support (Li et al., 2022). Given than lignin biosynthesis pathway crosstalk with other signaling pathways, including phytohormonal, post-transcriptional, post-translational, and epigenetic regulations (Novaković et al., 2018; Rao and Dixon, 2018; Dong and Lin, 2021), untangling this crosstalk will aid in identifying key hub targets for genetic or metabolic pathways manipulation for improved plant drought tolerance (Xie et al., 2018; Liu S. et al., 2023).

Besides lignin, other components such as glucuronoxylan have been implicated in plant drought stress response. More recently (Barbut et al., 2024), used RGB (red, green, and blue light) monitoring and hyperspectral imaging (HSI) techniques to reveal that the integrity of xylan backbone in SCW affects Arabidopsis response to drought stress. Compared to the wild-type, all Arabidopsis lines with impaired xylan integrity exhibited better survival, increased stomatal density and delayed growth inhibition under moderate drought stress conditions, although the magnitude of response was genotype-dependent (Barbut et al., 2024). Amongst the three xylan biosynthesis mutants *irx9*, *irx10* and *irx14* (*irx*, *irregular xylem*) and xylanase-expressing lines, *irx14* was the most drought-resistant, and the only genotype with increased lignin content and unchanged xylem conductivity (Barbut et al., 2024). These results suggest that modifying SCW integrity could be a potential strategy for developing drought-tolerant crop cultivars, although more studies are required to first understand the underlying molecular causes of SCW variation amongst genotypes. In support of these findings, engineered Arabidopsis plants with low xylan acetylation, and low lignin and xylan contents, have shown improved tolerance to severe drought stress than their wild-type counterparts (Yan et al., 2018a). Drought-tolerant plants displayed low leaf water loss rate and up-regulated expression of drought-responsive genes (*RD29A*, *RD29B*, *DREB2A*) under drought stress conditions, which did not occur under control conditions. Additionally, plants with low lignin content (as a result of expression of a 3-dehydroshikimate dehydratase, QsuB) exhibited a stronger response to ABA treatments, and accumulated more ABA in response to drought than the wild-type (Yan et al., 2018a). However, in plants with low xylan content or low xylan acetylation, drought tolerance was not related to ABA content or response differences, but, possibly, to increased galactose levels and increased sugar released under drought stress conditions (Yan et al., 2018a). Overall, these findings demonstrate the utility of enhancing plant drought tolerance through modification of lignin, xylans, and other SCW components.

### 4.5 Root suberization

The plant root endodermal cell layer essentially controls water and mineral nutrients uptake (Franke and Schreiber, 2007; Wang et al., 2019). Besides that, altering the extent of root cell wall suberization helps plants respond to abiotic and biotic stress (Chen et al., 2022; Kim and Sung, 2023). Suberized cells are generally waterproof, and act as barriers against pathogen invasion (Franke and Schreiber, 2007; Chen et al., 2022; Woolfson et al., 2022). Root suberin accumulation modulates salt and water transport, hence, is induced by salinity, cadmium (Cd), and ammonium stresses (Ranathunge et al., 2016; Wang et al., 2019) Suberization is also actuated under drought conditions (Henry et al., 2012), possibly via ABA (Kim and Sung, 2023), implying an essential role for suberin in preventing water loss (Chen et al., 2022). For instance, Arabidopsis root suberization has been shown to play crucial roles in minimizing water loss and NaCl uptake (de Silva et al., 2021). Prolonged water deficit stress increased the contents of suberin and suberin-related waxes in Wt Arabidopsis. Analysis of an Arabidopsis mutant collection showed that double mutant (*cyp86a1-1 cyp86b1-1*), with a significantly changed suberin composition and lamellae architecture, had elevated root peridermal cell water loss (de Silva et al., 2021). Equally, the triple mutant (*abcg2-1 abcg6-1 abcg20-1*), with changed suberin composition and lamellae structure, showed increased sensitivity to sodium, suggesting the essential role of suberin composition and lamellae architecture in minimizing peridermal cell water escape, and for limiting unrestrained sodium uptake, which assist plants to better tolerate drought and high-salinity stress conditions (de Silva et al., 2021).

During normal development, Arabidopsis Suberman (SUB) TF regulates root endodermal layer suberization. When transiently expressed in tobacco, SUB inducts several suberin-related genes, enhances suberin buildup, and lamellae deposition (Cohen et al., 2020). In drought-stressed rice roots, suberization of the endodermis has been shown to be increased, particularly in drought-tolerant genotypes, suggesting a critical water retention and drought tolerance strategy (Henry et al., 2012). Similarly, upregulated expression of suberin biosynthesis-related genes, and suberin lamellae accumulation is enriched in drought stressed barley roots (Kreszies et al., 2019), pointing to an essential plant drought stress tolerance mechanism (Kreszies et al., 2019). Meanwhile, overexpression of *MYB39* in Arabidopsis roots dramatically enhanced root suberization (Wang et al., 2020). ABA has been shown to elicit the activation of root suberization under osmotic stress (Wang et al., 2020). Besides, water deficit conditions significantly increased the induction of suberin biosynthesis-, ABA biosynthesis-, and aquaporins (AQPs)-related genes, and heightened lamellae suberization of the root endodermis in rice, suggesting that suberization, ABA metabolism and AQPs activity could dependently or independently regulate root drought tolerance in rice (Kim and Sung, 2023).

In tomato, which lacks endodermal suberin during normal development, it has been shown that a suberized exodermis is necessary for drought tolerance (Cantó-Pastor et al., 2024). Modulation of the tomato *MYB92* TF and Aliphatic Suberin Feruloyl Transferase (ASFT) enzyme could control exodermal suberin-related root responses to water deficit stress, and regulating the degree of exodermal suberization could be a new strategy for engineering drought tolerance in plants, whereas constitutive biosynthesis of endodermal suberin could aid in creating drought-tolerant plants, with enhanced CO_2_ sequestration capacity (Thompson, 2017). Several suberin metabolism–related genes (*KCS2*, *KCS20*, β-ketoacyl-CoA synthases, fatty acyl reductases, MYBs, etc.) and those of the phenylpropanoid biosynthesis pathway (compiled in (Vishwanath et al., 2015; Shukla et al., 2021; Chen et al., 2022; Woolfson et al., 2022; Cantó-Pastor et al., 2024)) can be targeted for modification, via overexpression, silencing, etc., to improve plant drought tolerance. Besides, agronomic interventions such as silicon-meditated suberization of roots can also aid in developing drought tolerance in plants such as rice and barley (Kreszies et al., 2020). However, tradeoffs or feedback regulations related to the modulation of suberin metabolism should be taken into account, since the outcomes of such modulation on plant growth, water and nutrient relations, plant-microbe interactions, and overall plant fitness are hardly predictable (Cohen et al., 2020; Woolfson et al., 2022; Cantó-Pastor et al., 2024).

### 4.6 Root cell wall remodeling related to root expansion and response to water extraction under drought

**Figure 5:**
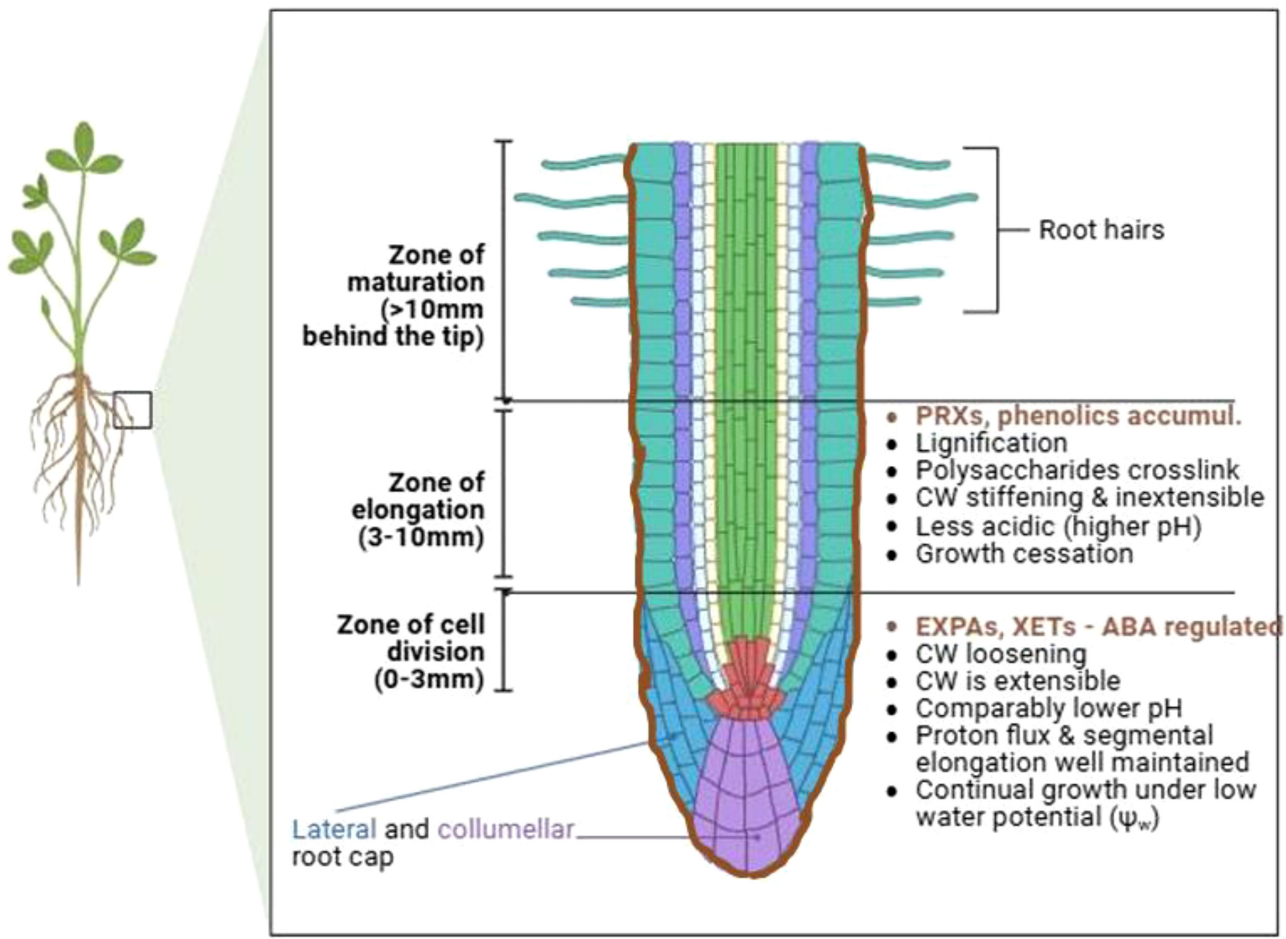
Differential cell wall (CW) responses occurring within different longitudinal root zones facilitate root adaptation to drought stress. Cell walls within the zone of cell division are loosened to allow continual growth even under conditions of low water potential, whereas those within the elongation zone are tightened (for instance, via lignification) to prioritize the growth of the apical segment and conservation of the extracted water within the root. Stiffening of the elongation zone also strengthens the root to facilitate root tip scavenging of deeper soil layers for water. For other parts labeling (from center to the outside), green, cream, sky-blue, purple, magenta, and brown represent the vascular cylinder, pericyte, endodermis, cortex, epidermis, and exodermis, respectively. EXPAs, expansins; XETs, xyloglucan endotransglucosylases; PRXs, peroxidases; ABA, abscisic acid; phenolics accum., phenolics accumulation. This illustration is based on (Wu and Cosgrove, 2000; Fan and Neumann, 2004; Fan et al., 2006) and others discussed in text. The figure was created using Biorender.com (https://app.biorender.com/).

Drought stress or water deficit is accompanied with decreased soil water potential. Under these conditions, plants tend to extend their roots into deeper soil layers in search for water (Takahashi et al., 2020). Thus, root system architecture adjustment represents a highly dynamic physical network that orchestrates plant access to a heterogeneous distribution of soil water (Feng et al., 2016). This process involves growth of the root (tips) and simultaneous preservation of extracted water within the root itself. Drought tolerance encompasses adaptations to growth under reduced water potential and the associated remodeling of the cell wall that permit growth occurrence under lower water contents. As such, drought tolerance results in both loosening and tightening of the cell wall, in different root zones (Moore et al., 2008). Tissues that are necessary to maintain in a growth-ready state are loosened, whilst non-essential tissues are tightened to prioritize continual growth of vital growing points such as the root apex even under lower turgor pressure (Wu and Cosgrove, 2000; Moore et al., 2008) (**Figure 5**). To permit cell wall growth, the threshold turgor pressure in these growing points is altered.

Studies on maize root responses to drought stress treatment (Wu et al., 1994; Wu et al., 1996; Wu and Cosgrove, 2000) have been the most revealing about drought effects on cell wall structure and properties. Differential cell wall responses to drought stress treatment have been observed in different root zones (Voothuluru et al., 2016). On one hand, the cell walls within the root apical zone (0-3 mm behind the tip) are kept in a flexible/extensible state to permit slow tissue growth within this region even under low water potentials. On the other hand, cell walls within the elongation zone (3-9 mm behind the tip) are stiffened or made inextensible to halt further growth within that region (Fan and Neumann, 2004; Fan et al., 2006) (**Figure 5**). Different mechanisms have been shown to underpin these differential cell wall responses (loosening and stiffening) within these two different root zones. First, drought stress results in spatial difference in cell wall-associated pH between the two root zones, with the growing tip having a comparably lower pH to the elongation zone (Fan and Neumann, 2004). This observation resonates well with the canonical acid growth expansion theory, whereby auxin phosphorylation and activation of PM proton pumps (H^+^-ATPase) induces cell wall (apoplastic) acidification, triggers expansins and cell wall relaxation (McQueen-Mason et al., 1992; Wu et al., 1996; Fan and Neumann, 2004), which may promote organ expansion (Lin et al., 2021). Secondly, water channels are also believed to allow preferential water conveyance to the root tip cell walls, aiding cell wall loosening in that region (Moore et al., 2008). Thirdly, expansins and XETs may also be involved in cell wall loosening within the root growing tip, possibly regulated by ABA (Wu et al., 1994; Wu et al., 1996). Expansins modulate cell wall loosening via disruption of hydrogen bonding in the wall without compromising polysaccharide backbones (Cosgrove, 2024). XETs tend to increase their activity within the cell wall loosening (apical 6mm) region under low water potentials, as regulated by ABA (Wu et al., 1994). Meanwhile, Arabidopsis root responses to salinity have been shown to rely on pectin modification (mediated by PMEs) and cell wall sensing, whereby these responses partially require the functionality of FERONIA alone or HERKULES 1/THESEUS1 (HERK1/THE1) to diminish salt effects (Gigli-Bisceglia et al., 2022). Considering that salinity and drought are both osmotic stresses, these results may point to possible involvement of pectin remodeling in root responses to drought stress (Piro et al., 2003; Leucci et al., 2008); this will need further experimental investigation.

The accumulation of phenolics and lignin within the root elongation region, and their subsequent crosslinking to the cell wall polysaccharides, as mediated by peroxidases, stiffens the cell wall (Fan et al., 2006), whereas lignin accumulation induces removal of water from the wall, thus causing the cell wall to be inextensible and growth within the root elongation zone to cease (Fan and Neumann, 2004; Moore et al., 2008). This has been supported by the observation that lignin biosynthesis-related enzymes increase their activities with drought stress in the root elongation zone (Fan and Neumann, 2004). Taken together, differential cell wall responses (loosening and tightening) within the two different root zones facilitate for root adaptation to drought stress, by orchestrating continued growth of the root tip under water deficit conditions, and simultaneous preservation of the extracted water within the root itself. Besides, stiffening of the root elongation zone strengthens the growing tip to bore deeper through the “drier” soil layers in search of water.

## 5 Phytohormonal regulation of cell wall remodeling-mediated drought tolerance

**Figure 6:**
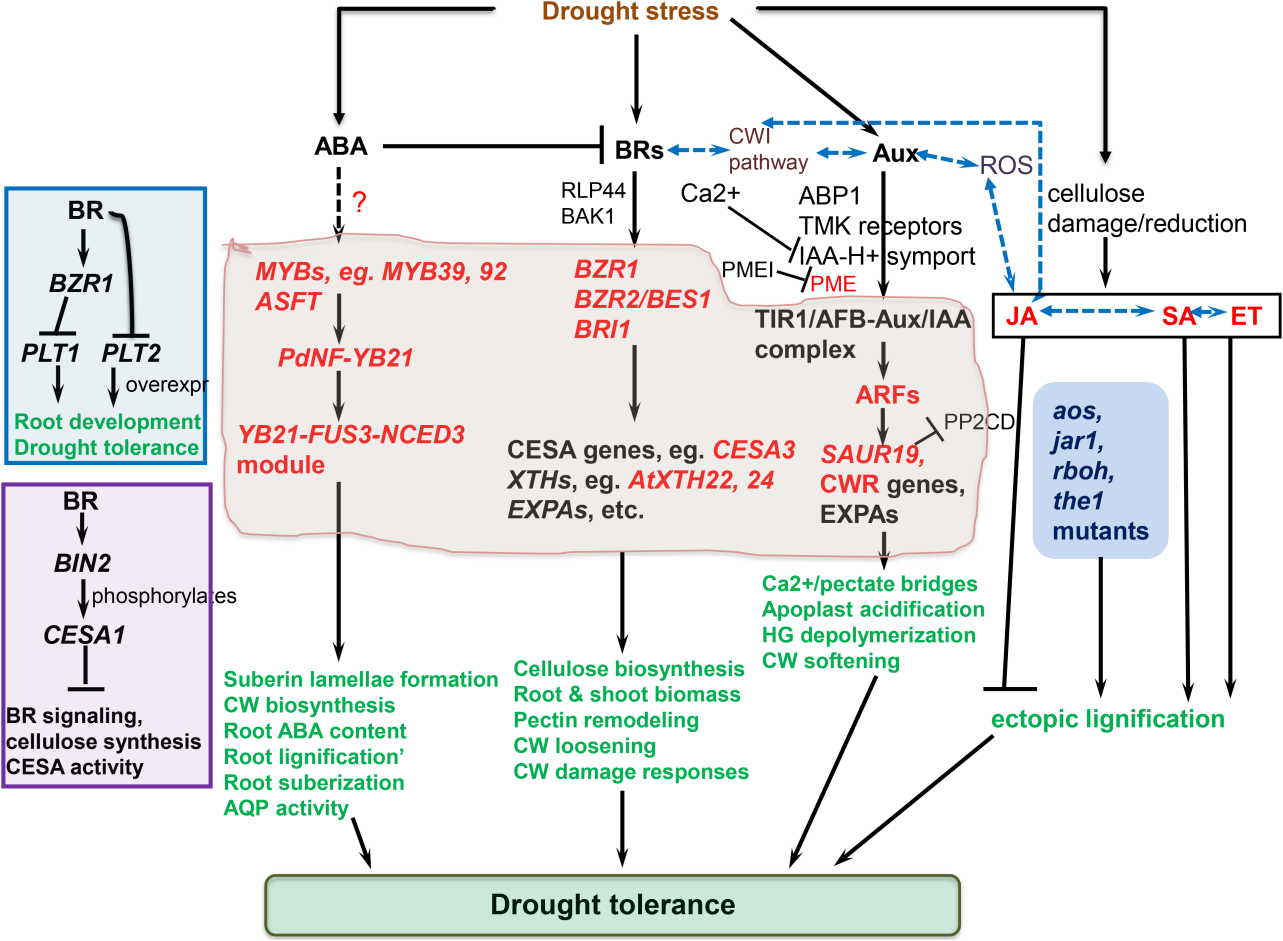
Phytohormonal regulation of cell wall remodeling (CWR) mechanisms related to drought stress tolerance discussed in this review. *BRASSINAZOLE-RESISTANT 1/2 (*BZR1/2) or *BRASSINOSTEROID-INSENSITIVE* (BRI)-mediated brassinosteroid (BR) signaling regulates the actuation of several CWR-related genes and processes. Abscisic acid (ABA) signaling and MYB transcription factors modulate auxin-regulated root growth, root suberization and/or lignification, and drought tolerance. Auxin signaling modulates expression of CWR enzymes regulating pectin properties. Stress-triggered cellulose damage or reduction elevates jasmonic acid, salicylic acid and ethylene levels, alters cell wall composition and architecture, and promotes ectopic lignin deposition (ELD). Dashed black arrow and question mark imply that the signaling pathway needs further clarification. Encasing in the center represent nuclear processes. Blue dashed arrows (not exhaustive) denote crosstalks, red font implies upregulated expression or elevated level, whilst green font implies improved physiological response/trait. NF-YB21, nuclear factor YB21; PdFUS3, a B3 domain transcription factor; ASFT, ALIPHATIC SUBERIN FERULOYL TRANSFERASE; NCED3, NINE-CIS-EPOXYCAROTENOID DIOXYGENASE 3; CWI pathway, cell wall integrity pathway; RLP44, receptor like protein 44; ROS, reactive oxygen species; BRI1, *BRASSINOSTEROID-INSENSITIVE 1*; BIN2, *BRASSINOSTEROID INSENSITIVE 2*; BES1, BRI1-EMS-Suppressor 1; BAK1, *BRI1-associated receptor kinase 1;* PLT1/PLT2, *PLETHORA 1/2*; *CESA1*, *cellulose synthase A1*; EXPAs, expansin As; XTHs, xyloglucan endotransglucosylases/hydrolases; PME, pectin methyltransferase; PMEI, PME inhibitor; SAUR19, SMALL AUXIN UP RNA 19; ARFs, auxin-responsive factors; AQP activity, aquaporin activity; *aos, allene oxide synthase; jar1, jasmonic acid resistant 1*; *rboh, respiratory burst oxidase homologues; the1, theseus1* receptor-like kinase; Main figure is based on (Zhou et al., 2020) (for ABA), (Rao and Dixon, 2017; Novaković et al., 2018; Tiika et al., 2021) (for BRs), (Majda and Robert, 2018; Jobert et al., 2023) (for auxins), (Denness et al., 2011) (for jasmonic acid), and others discussed in text. Top left insert is based on (Zhao et al., 2023), whilst bottom left insert is based on (Sánchez-Rodríguez et al., 2017).

Phytohormones regulate cell wall properties, growth, development, and cell wall stress signaling, as well as transcriptional output of some genes encoding cell-wall remodeling enzymes. In turn, the cell wall modulates the homeostasis of these phytohormones (Wolf et al., 2012), creating an interplay essential for stress response (Jobert et al., 2023). For instance, ABA, as already briefly highlighted above, ABA regulates suberization, AQP activity and drought tolerance in rice roots (Kim and Sung, 2023). Besides, endodermal ABA signaling mediates suberization responses to nutrient stress in Arabidopsis roots (Barberon et al., 2016). ABA signaling transduction mediates suberin lamellae formation (Wang et al., 2020), and cell-wall biosynthesis (Dalal et al., 2018) in drought-exposed roots, as has been observed in rice (Henry et al., 2012), wheat (Dalal et al., 2018), Arabidopsis (Wang et al., 2020), and barley (Kreszies et al., 2019). Similarly, ABA-treated wheat exhibited increased root suberin lamellae, whereas Arabidopsis showed accelerated suberin deposition under drought conditions (Grünhofer et al., 2021), suggesting that drought induces suberin biosynthesis, and ABA-signaling may coordinate root suberization and drought tolerance in such species (Wang et al., 2020; Kim et al., 2022) (**Figure 6** **).** However, the exact mechanism underlying ABA-mediated root suberization under varying water potentials remains unexplored (Kim and Sung, 2023).

BR-mediated modulation of stress-triggered cellulose synthesis seems to be evolutionarily conserved in certain species (Kesten et al., 2017). For instance, increased levels of *BRI1*, *CESA3*, and other BR signaling-associated genes were observed in progenitor species *Agropyron elongatum* than in common/cultivated wheat genotypes in response to water stress (Placido et al., 2013). When introgressed into cultivated genotype, the wheat translocation line exhibited enhanced drought tolerance and greater root and shoot biomass in comparison with the control under stress (Placido et al., 2013), suggesting that the enhanced BR-signaling pathway of *Agropyron elongatum* could contribute to its higher drought stress adaptation, via improved root and shoot biomass, that possibly facilitate enhanced water extraction under stress (Placido et al., 2013; Rao and Dixon, 2017). Additionally, BRs mediate several cell wall remodeling processes, including cell wall damage responses, cell wall signaling, CWI maintenance, cell wall loosening, cellulose deposition, lignin accumulation, pectin modification, etc. (Zurek and Clouse, 1994; Rao and Dixon, 2017; Novaković et al., 2018; Rao and Dixon, 2018) (**Figure 6**). BR hormone signaling pathway also modulates the induction of several cell wall-associated genes (Wolf et al., 2012). In Arabidopsis, BR-activated TF BZR1 and its homology BZR2/BES1 directly bind to the promoter regions (such as the CANNTG-E motifs) of numerous cell wall-associated and BR-responsive genes such as the cellulose synthase A (CeSA) genes (Xie et al., 2011; Li et al., 2018), and NAC and MYB TFs linked to lignin biosynthesis pathways (Zhao and Dixon, 2011; Rao and Dixon, 2017; Rao and Dixon, 2018). However, Brassinosteroid Insensitive 2 (BIN2), a protein kinase, directly phosphorylates Arabidopsis *CESA1*, negatively regulating BR signaling, CESA activity and cellulose biosynthesis (Sánchez-Rodríguez et al., 2017) (Bottom left insert of **Figure 6**). CESA genes, such as *CESA1*, are transcriptionally and post-transcriptionally modulated via the BR-mediated signaling to regulate cellulose synthesis in Arabidopsis (Novaković et al., 2018). Besides, BRs regulate the expression of genes encoding cell wall loosening EXPAs and XTHs (Zurek and Clouse, 1994; Novaković et al., 2018). XTHs (eg., *AtXTH22*, *AtXTH24*, etc.) and EXPA genes have been shown to be considerably up-regulated by BR treatment in Arabidopsis and soybean (Zurek and Clouse, 1994; He et al., 2003), and in response to salinity and drought stress in halophytic plant *Salicornia europaea* (Tiika et al., 2021).

BRs also modulate stress-induced pectin remodeling. In particular, BR signaling crucially modulate cell wall modifying proteins, including PMEs (Wolf et al., 2012), whereupon stress-induced feedback mechanism from the cell wall potentiates the output of the BR pathway to ensure cell wall homeostasis and CWI in Arabidopsis (Wolf et al., 2012; Novaković et al., 2018). More recently, BR signaling has been shown to modulate root growth and drought tolerance in Arabidopsis, by repressing the expression of transcription factors *PLETHORA 1* and *2* (*PLT1* and *PLT2*, involved in root development, including apical meristem embryonic pattern formation (Aida et al., 2004)) (Zhao et al., 2023) (Top left insert of **Figure 6**). *PLT1*-OE and *PLT2*-OE transgenic Arabidopsis exhibited higher water deficit tolerance as compared to their Wt counterparts. Additionally, both *in vivo* and *in vitro* experiments have shown *BZR1* to bind to *PLT1* promoter region and hinder its transcription activation (Zhao et al., 2023). Further, BR signaling regulated *PLT1* and *PLT2* expression in root development, and *PLT2* partly rescued the drought sensitivity of *bes1-D* mutant (Zhao et al., 2023). It might be probable that these genes could play other essential roles in cell wall remodeling processes linked to drought tolerance, and will need further exploration.

Auxin (Aux) is another phytohormone that regulate the expression and activities of cell wall remodeling-involved enzymes, including EXPAs, PMEs, PODs, etc (Cosgrove, 2015; Nafisi et al., 2015; Cosgrove, 2016b). These enzymes crucially mediate cross-linking of lignins, pectins, and proteins, resultantly influencing stress-induced cell elongation (Passardi et al., 2004; Passardi et al., 2006; Liu X. et al., 2021). Essentially, Aux signaling potentiates the transcriptional output of several pectin remodeling related enzymes-encoding genes, their local activity, and spatio-temporal distribution or modulation of pectin (Jobert et al., 2023). Using atomic force microscopy (AFM) (Braybrook and Peaucelle, 2013), established a link between Aux signaling and PME activity that modulates cell wall biochemical and mechanical changes necessary for organ formation in the Arabidopsis shoot apical meristematic region. Auxin also triggers low pH, which activates PME and hinders PMEI (see (Majda and Robert, 2018)). Besides, indole-3-acetic acid (IAA) orchestrate several plant growth and development processes (Sánchez-Rodríguez et al., 2010; Zhao, 2010), including the canonical acid growth cell expansion theory (McQueen-Mason et al., 1992). Auxin phosphorylates and activates PM proton pumps (H^+^-ATPase) to control apoplastic pH, which triggers expansins and relaxes the cell wall (McQueen-Mason et al., 1992). The transmembrane kinases also directly interact/phosphorylate PM H^+^-ATPases, evoking their activation, and inducing cell-wall (apoplastic) acidification, and hypocotyl cell elongation in Arabidopsis (Lin et al., 2021). Auxin acidification modulates the relaxation of cell wall matrix due to loosening of the binding between cell wall polysaccharides, as controlled by XTHs and cellulases. The expression of these XTHs and cellulases is upregulated by auxin. When cellulase activity is increased by auxin, the load-bearing hemicellulose chains are cleaved, causing modification of interactions between polysaccharides and other cell wall components. This leads to eventual cell wall loosening and extensibility (nicely reviewed in (Majda and Robert, 2018)). However, how these hormonal crosstalks regulate drought stress response and adaptation in crops is yet to be fully understood. It will be interesting to understand, and/or quantify, how cells balance growth/development- and stress-response-related portions of cell wall modifications.

Other phytohormones, including jasmonic acid (JA), salicylic acid (SA), ethylene, etc., also participate in cell wall remodeling-mediated abiotic stress response (Ellis et al., 2002; Novaković et al., 2018), and defense ( (Nafisi et al., 2015). The levels of these hormones are elevated in response to stress-induced cellulose damage or reduction, leading to cell wall compositional and mechanical shifts, including ectopic lignification (Kesten et al., 2017) (**Figure 6**). In certain situations, these phytohormone (eg., BR and JA) signaling pathways intricately interact with ROS and CWI pathways to regulate stress response and adaptation (Nafisi et al., 2015; Novaković et al., 2018). For instance, on one hand, JA inhibits stress-induced cell-wall-remodeling-related ectopic lignin accumulation in Arabidopsis (Denness et al., 2011). Mutants (eg., a*llene oxide synthase*, *aos;* and *jasmonic acid resistant 1*, *jar1*) with stifled JA production exhibit elevated lignin accumulation prompted by the damage to the cell wall (Denness et al., 2011). On the other hand, mutants with repressed ROS generation and CWI detection (eg., *rboh* and *the1*), exhibit elevated contents of ectopic lignin caused by the damage to the cell wall (Denness et al., 2011), revealing the important crosstalk coupling JA, ROS and CWI pathways as necessary for cell wall damage-triggered responses. Decoding such crosstalk helps in clarifying how drought tolerance is regulated by each or combination of the cell-wall remodeling-involved components or pathways, and could facilitate for appropriate cell wall adjustments, or metabolic engineering for drought tolerance in crop plants (Neumann, 1995; Liu S. et al., 2023).

## 6 Advances in plant cell wall imaging are driving cell wall remodeling investigations

**Figure 7:**
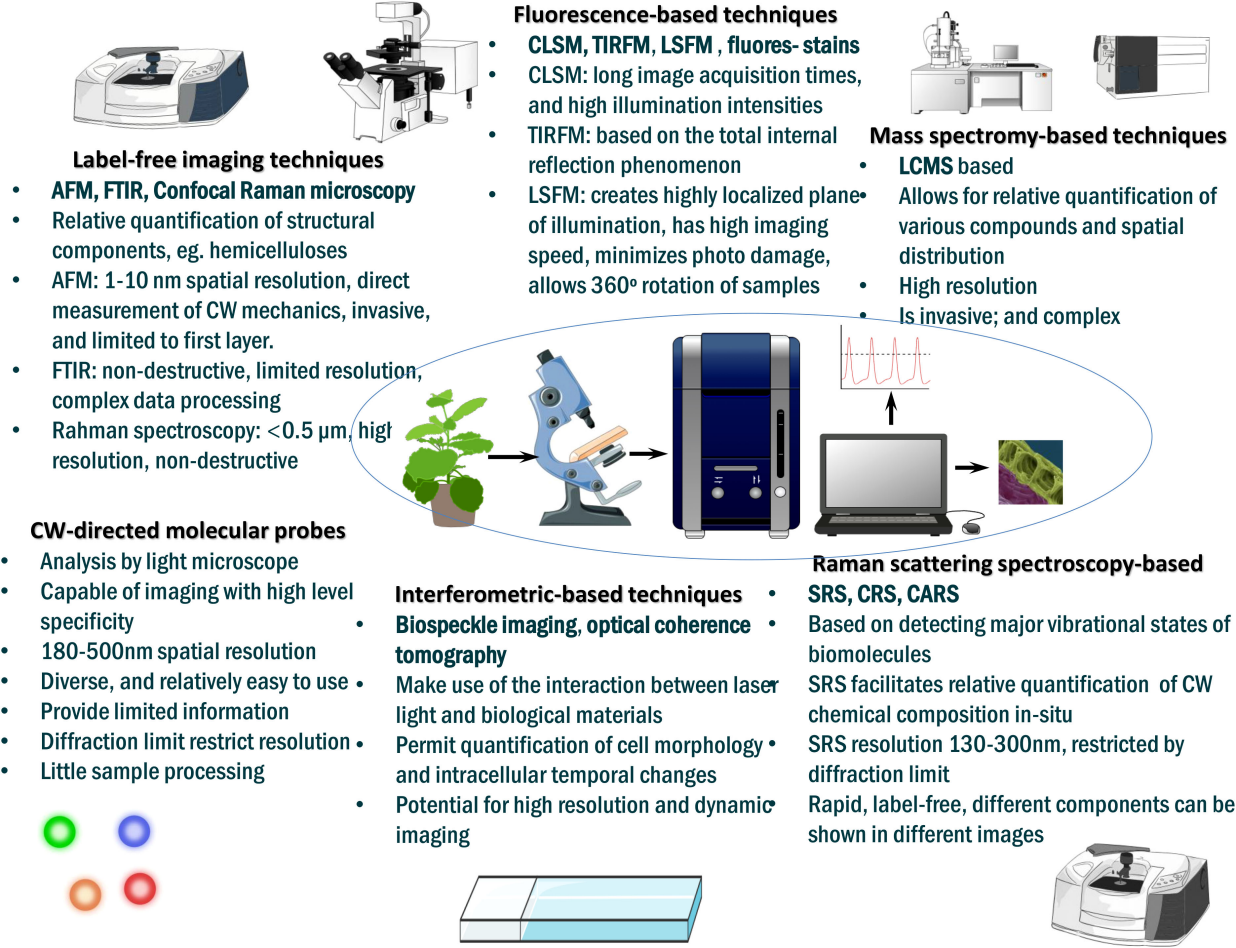
Summary of advanced plant cell wall imaging techniques (not exhaustive). CW, cell wall; AFM, atomic force microscopy; CLSM, confocal laser-scanning microscopy; CRS, coherent Raman scattering microscopy; CARS, coherent anti-Stokes Raman scattering (CARS) microscopy; FTIR, Fourier-transform infrared spectroscopy; LCMS, liquid chromatography-mass spectrometry; LSFM, light sheet fluorescent microscopy; SRS, stimulated Raman scattering microscopy; TIRFM, total internal reflection fluorescence microscopy. For detailed reviews, visit (Zhao et al., 2019; Mateu et al., 2020; DeVree et al., 2021; Xu et al., 2021; Alonso Baez and Bacete, 2023; Piccinini et al., 2024).

Over the past two decades, a repertoire of plant cell imaging techniques has been developed to aid the functional visualization of plant cell structural architecture and biosynthetic processes at different spatiotemporal scales, offering a better understanding of how the plant cell wall functions under different conditions (Higashiyama et al., 2021; Xu et al., 2021) (**Figure 7**). Particularly, the deployment of different microscopy models, including polarized light microscopy (Radosavljević et al., 2021), scanning electron microscopy (SEM) (Fromm et al., 2003; Wightman, 2022), field emission scanning electron microscopy (FESEM) (Zheng et al., 2017), etc., revolutionized plant cell/tissue/organ imaging, and significantly enhanced visualization and quantification of subtle cell wall compositional and morphological changes, which helped in resolving some complex biological questions that could not be answered by conventional biochemical assays (Komis et al., 2018; Cui et al., 2023).

Fluorescence-based techniques, including confocal laser-scanning microscopy, total internal reflection fluorescence microscopy, light sheet fluorescent microscopy, etc., have provided insights into plant cell wall composition, structure, and biosynthetic machineries such as lignin and pectin cross-linking (reviewed in (Tobimatsu et al., 2013; Zeng et al., 2017; Kitin et al., 2020; DeVree et al., 2021)). Additionally, several histochemical and immunolabeling tools can be used for direct labeling, targeted analyses, and/or real-time visualization of dynamics of cell wall polysaccharides or other non-polysaccharide components, thereby offering new ways to explore the physiological mechanisms of cell-wall remodeling (Barnes and Anderson, 2018; Rydahl et al., 2018; Voiniciuc et al., 2018; DeVree et al., 2021). These histochemical and immunolabeling techniques include the monoclonal antibodies, small-molecule probes and fluorescently tagged monolignols (detailed in (Rydahl et al., 2018; Ursache et al., 2018)). For instance, 2F4 monoclonal antibody has been successfully used to detect how pectin HGs, one of the major plant cell wall components, can form pectate complexes with divalent Ca^2+^ ions (egg boxes) when GalA residues are blockwise de-esterified (Liners et al., 1989), since this mechanism crucially regulate wall biomechanics and mediates cell-cell bonding (Du et al., 2022). Meanwhile, turgor pressure changes within guard cells during drought stress regulate stomatal opening and closing (Auler et al., 2022), and these are governed by biochemical and mechanical shifts of the GCWs (Amsbury et al., 2016). A histological technique, monoclonal antibody LM20 – with binding affinity to highly methylesterified HGs, has been used to differentiate guard cell wall components from adjacent epidermal cells. Based on the variations in immunofluorescence, Arabidopsis GCWs are distinguished by low level of methylated pectins (Amsbury et al., 2016), thereby providing an effective way for identifying GCW components and understanding GCW modifications during drought stress (Alonso Baez and Bacete, 2023). Recently, an inventive oligogalacturonide-derived molecular probe (modeled on Ca^2+^-dependent attachment of fluorescently-labeled oligogalacturonides to endogenous de-esterified HGs) was developed to competently monitor HG crosslinking dynamics in elongating pollen tube- and root hair cell walls of Arabidopsis in real-time (Mravec et al., 2017), thereby further clarifying the mechanisms by which cell walls are remodeled. Equally (Besten et al., 2025), developed CarboTag, a modular approach for live and functional fluorescence imaging of plant cell walls. It is based on a small molecular motif, a pyridine boronic acid, that undergoes high-affinity binding with diols in the plant cell wall (Besten et al., 2025). CarboTag enabled the authors to develop several cell wall imaging probes (in different colors) for multiplexing, and novel functional reporters for live and quantitative imaging of key cell wall properties such as network porosity, cell wall pH, and the presence of ROS (Besten et al., 2025). This technique opens the way for dynamic and quantitative mapping of cell wall responses to various perturbations, including abiotic stress, at subcellular resolution. Moreover, it is applicable to diverse plant and brown algae species without the need for genetic manipulation of the organism of interest (Besten et al., 2025). Besides, fluorescent proteins (FP) can be conveniently tagged to cell wall-specific fluorophores, permitting simultaneous visualization of FP-labelled proteins and cell wall components (Ursache et al., 2018; Voiniciuc et al., 2018; DeVree et al., 2021). Further, coupling cell wall-directed molecular probes to light microscope-based analysis facilitates high-level-specificity imaging, minimal sample processing, and real time parsing of cell wall assemblies, thereby “simplifying” our understanding of plant cell wall architecture and stress-related response mechanisms (DeVree et al., 2021).

Meanwhile, high-resolution label-free imaging techniques, including AFM (Zhang et al., 2016; Song et al., 2020; Pu et al., 2022; Pu et al., 2025), Fourier-transformed infrared (FTIR) microspectroscopy (Smith-Moritz et al., 2015), hyperspectral imaging (HSI) (Sarić et al., 2022), transmission electron microscopy (TEM) (Fromm et al., 2003), confocal Raman microscropy (µ-Raman) (Gierlinger et al., 2012), etc., now enable direct and powerful visualization, and revelation of molecular and native chemical composition, as well as structural architecture of cell walls (Barnes and Anderson, 2018; Zhao et al., 2019; Alonso Baez and Bacete, 2023). For instance, AFM technique has been used to directly image PCWs and SCWs from fresh maize tissues under near-native conditions (Song et al., 2020). By analyzing cellulose structure in these different cell wall types, individual microfibrils and bundles in these cell walls were measured at nanometer scale, which helped to parse the different mechanisms of cellulose biosynthesis in PCWs and SCWs (Song et al., 2020). Similarly (Braybrook and Peaucelle, 2013), used AFM to reveal a feedback loop between Aux signaling and pectin-mediated cell wall remodeling that underpins organ formation and development in Arabidopsis (Braybrook and Peaucelle, 2013). The latest AFM-based infrared/Raman spectroscopy allows for *in situ* imaging of the multidimensional structure of the cell wall, revealing the mechanical characteristics of plant tissues or single cells, and specific single-molecule recognition of cell wall-related enzymes (Pu et al., 2025). As already discussed above, stomatal opening determines drought resistance, and guard cell walls have a crucial role in this process (Pirasteh-Anosheh et al., 2016). Since FTIR microspectroscopic imaging technique allows for a detailed analysis of the biochemical composition of specific cell walls *in situ*, and has already been used to classify cell wall mutants (Mouille et al., 2003), it could be a suitable fit for in-depth analysis of the composition and orientation of guard cell wall (GCW) polymers during drought stress (Alonso Baez and Bacete, 2023).

Hyperspectral imaging (HSI) is a noninvasive, label-free, rapid, and high-throughput plant phenotyping (HTPP) technique that acquires and processes both spectral (λ) and spatial (*x*, *y*) information and merges these into a 3D data matrix, referred to as ‘hyperspectral data cube’, with HSI sensors capturing information from the entire wavelength spectrum (UV, near-infrared, visible, and short-wave infrared in the 250–2500 nm regions) (Sarić et al., 2022). Moreover, HSI systems have a finer resolution (<5 nm) with tens to hundreds of spectral bands in a continuous range (Mehta et al., 2018). In comparison, RGB imaging collects light interacting with a sample at only three distinct wavelengths (red – 630 nm, green – 545 nm, and blue 435 nm) and the information on the location (spatial information) from which the light is being collected (Mehta et al., 2018). The additional spectral information provided by HSI facilitates for more accurate analysis and understanding of micro and nanoscale properties of the plant cell walls that are not detectable using RGB imaging (Mehta et al., 2018). HSI is a powerful and important tool for plant cell wall visualization and analysis, and has been applied in determining plant traits (eg., root traits), and detecting plant abiotic and biotic stress (eg., early phases of plant disease) responses (Sarić et al., 2022). For instance (Charrier et al., 2021), used a multimodal scattering near-field optical microscopy (SNOM) technique for *in-situ* HSI of poplar wood material. This technique facilitated determination of nanoscale properties of PCWs by correlating the local optical, chemical, and mechanical properties at a spatial resolution of 20 nm, which enabled monitoring of the delignification process and different lignin acetylation yields in relation to their structure and location in the PCW of poplar wood (Charrier et al., 2021). It is possible that this same technique can equally be applied to monitoring drought stress-induced cell wall modifications such as acetylation or demethylesterification of pectin. Meanwhile (Barbut et al., 2024), combined RGB and HSI techniques to reveal that the integrity of xylan backbone in SCW affects Arabidopsis response to drought stress. Coupling such advanced techniques to single cell analysis and machine learning approaches now permits for real-time *in vivo* monitoring of plant stress response, such as stress-related H_2_O_2_ signaling, and accurate differentiation of different types of stress, ultimately enhancing our understanding of the mechanisms of plant stress signaling (Hu et al., 2025).

TEM is a high-resolution microscopic technique that employs highly energetic electrons for the *in situ* analysis of the ultrastructure (at atomic scale) and direct visualization of the nanoscale morphologies of cellulosic materials (especially the “nanocelluloses” – nanofibers and nanocrystals) through a highly magnified image (Ogawa and Putaux, 2019). Now, time-resolved *in situ* TEM enables unprecedented understanding of the ultrastructure of materials and how structure is related to properties and function (Alcorn et al., 2023). Already, TEM technique has been applied in ultrastructural and immunohistological investigations of plant viral pathogens (Zechmann and Zellnig, 2009), and can be deployed for real-time assessment of cell wall remodeling dynamics during drought stress. When coupled to molecular probes, ie., colloidal gold-conjugated antibodies (to form immunogold TEM), TEM immunolocalizes polysaccharides across the cell wall thickness at much higher resolution (Sun et al., 2017). Additionally, immunogold TEM offers information on the relative abundances of target polysaccharides in a cell wall (Liners and Van Cutsem, 1992), thereby providing utility for analyzing the composition and distribution of cell wall polysaccharides at an ultrastructural level (Sun et al., 2017; Majda, 2019).

The latest cutting-edge Raman microscopy (µ-Raman), which integrates the chemical analysis technique, Raman spectroscopy (dependent on inelastic or Raman scattering of monochromatic laser light interacting with biomolecules), with a traditional light microscope (Gierlinger et al., 2012), is a non-destructive label-free technique that facilitates *in-situ* analysis of chemical composition and direct visualization of the structures of cell wall components such as pectins at micrometer (<0.5 μm) and nano-scale levels (Mateu et al., 2020). µ-Raman based approaches, including the coherent Raman scattering (CRS) microscopy, coherent anti-Stokes Raman scattering (CARS) microscopy, and stimulated Raman scattering (SRS) microscopy (Mateu et al., 2020; Xu et al., 2021), are modelled on detecting biomolecules` major vibrational states (Kumamoto et al., 2018), thereby offering label-free dynamics, rapid, high-specificity, and quantitative microanalysis of cell walls chemical compositions in their native states (Cui et al., 2023). µ-Raman is used in live imaging of cell wall biosynthetic processes (Xu et al., 2021) and cell wall lignification (Schmidt et al., 2010). Thus, µ-Raman spectroscopy is important for elucidating plant cell wall cross-linking chemistry and polymeric architecture (Zeng et al., 2017), improving our fundamental understanding of the dynamic processes involved in stress-induced cell wall remodeling (Zhao et al., 2019). When integrated to super-resolution microscopy, such as structured illumination microscopy, photoactivation localization microscopy, etc., and real-time analyses of dynamic cell wall structural changes (through use of light-sheet fluorescence microscopy), these label-free imaging technologies fine-tune the molecular characterization, and enhance our knowledge of temporal cell wall structural and compositional shifts under stress conditions (Komis et al., 2018; Zhao et al., 2019). This may help us to deconstruct individual constituents of CWI, phytohormonal, and ROS signaling networks, and to decipher cell wall regulation by their crosstalk (Novaković et al., 2018). Meanwhile, the surface-enhanced Raman scattering (SERS)-based nanoprobe is used for the real-time *in vivo* monitoring of multiple endogenous stress signaling molecules in plants (Son et al., 2023). The nanosensor is placed in the intercellular space, and is optically active in the near-infrared region (785 nm), enabling it to evade interferences from plant autofluorescence (Son et al., 2023). The method has been successfully used to detect abiotic and biotic stress-related molecules, including phytohormone salicylic acid, extracellular adenosine triphosphate (ATP), cruciferous phytoalexin, and glutathione in *Nasturtium officinale*, bread wheat, and barley species, signposting the possible onset of plant stress, including disease (Son et al., 2023). This paves the way for the possibility of monitoring the commencement or early processes of stress in plants, including the key molecules involved.

Furthermore, the latest label-free interferometric-based technologies, such as biospeckle imaging, optical coherence tomography, etc., can harness the interaction of laser light with biological materials to deduce (from interference patterns) important structural and activity information, permitting measurement of cell structure and temporal intracellular changes (Ebrahimi et al., 2023). For instance, laser speckle contrast imaging has been used to monitor the dynamic changes occurring during the growth of the apical root region of beetroot (*Beta vulgaris*) (Schott et al., 2022). It is possible that this technique can be harnessed for *in situ* monitoring of the physiological processes, and in particular the cell wall remodeling mechanisms occurring within the root apical region under drought stress. Besides, interferometric-based technologies have shown high utility in determining seed germination capacity, plant growth, plant disease, etc., and are envisaged to facilitate high-resolution and dynamic imaging of plant cell walls at various spatiotemporal scales (Ebrahimi et al., 2023). This will enhance our knowledge of abiotic stress-triggered cell wall modifications and crosstalk among multiple interconnected pathways.

Despite these recent advances, however, understanding drought stress-induced cell wall remodeling still remain conceptually and technically challenging. This is because of several reasons. First, disentangling the complex crosstalk among CWI maintenance, cell wall stress sensing and signaling, phytohormonal signaling, and ROS production mechanisms remains cumbersome, especially deconstruction of the individual components constituting each of those networks, and grasping how cell wall stress sensors decode or differentiate specific abiotic stress-induced cell wall modifications to institute narrowly targeted responses (Kumar et al., 2016; Novaković et al., 2018). Secondly, the non-quantitative nature of the data output of some of the low-end biochemical and cytological exploration techniques still in use, and their inability to monitor dynamic changes in plant development or biomass transformation limit their overall effectiveness (Zhao et al., 2019; Xu et al., 2021). Fortunately, the recent cutting-edge imaging techniques highlighted above are countering some of these shortcomings. Furthermore, despite the central role of cell wall-localized proteins like EXPAs, XTHs, or PMEs in assembly and spatiotemporal cell wall control, their precise localization or movement within the cell wall, remains largely unresolved (DeVree et al., 2021). Designing specific probes targeting these cell wall proteins will be crucial in understanding the connection between the cell wall protein activity, localization, and formation of cell wall microdomains, and whether engineering of these proteins could be useful in designing cell walls with desired characteristics to enhance plant stress tolerance (DeVree et al., 2021). Going forward, coupling these latest high-resolution imaging techniques to high-sensitivity mass spectromy-based analytical and spatially resolved singe-cell omics approaches (Zhang H. et al., 2023), and advanced data analysis (eg., multivariate) methods (Moore et al., 2020) will immensely improve our understanding of the cell wall composition, structure, biosynthetic machineries, function, and regulation at spatial and time-gated scales (DeVree et al., 2021). Besides, synthetic biology (Shelake et al., 2022) and genome-editing approaches (Zafar et al., 2020) are rapidly “simplifying” deconstruction of complex networks, and facilitating metabolic pathway engineering for enhanced stress tolerance (Liu S. et al., 2023). We anticipate progressive improvement in these technologies to facilitate appropriate cell wall modifications (for instance, via genetic, or in-planta biosynthetic pathways engineering (Yoshida et al., 2021)) necessary for developing more drought-tolerant crop plants tailor-fit for the new climatic environments.

## 7 Conclusion

**Figure 8:**
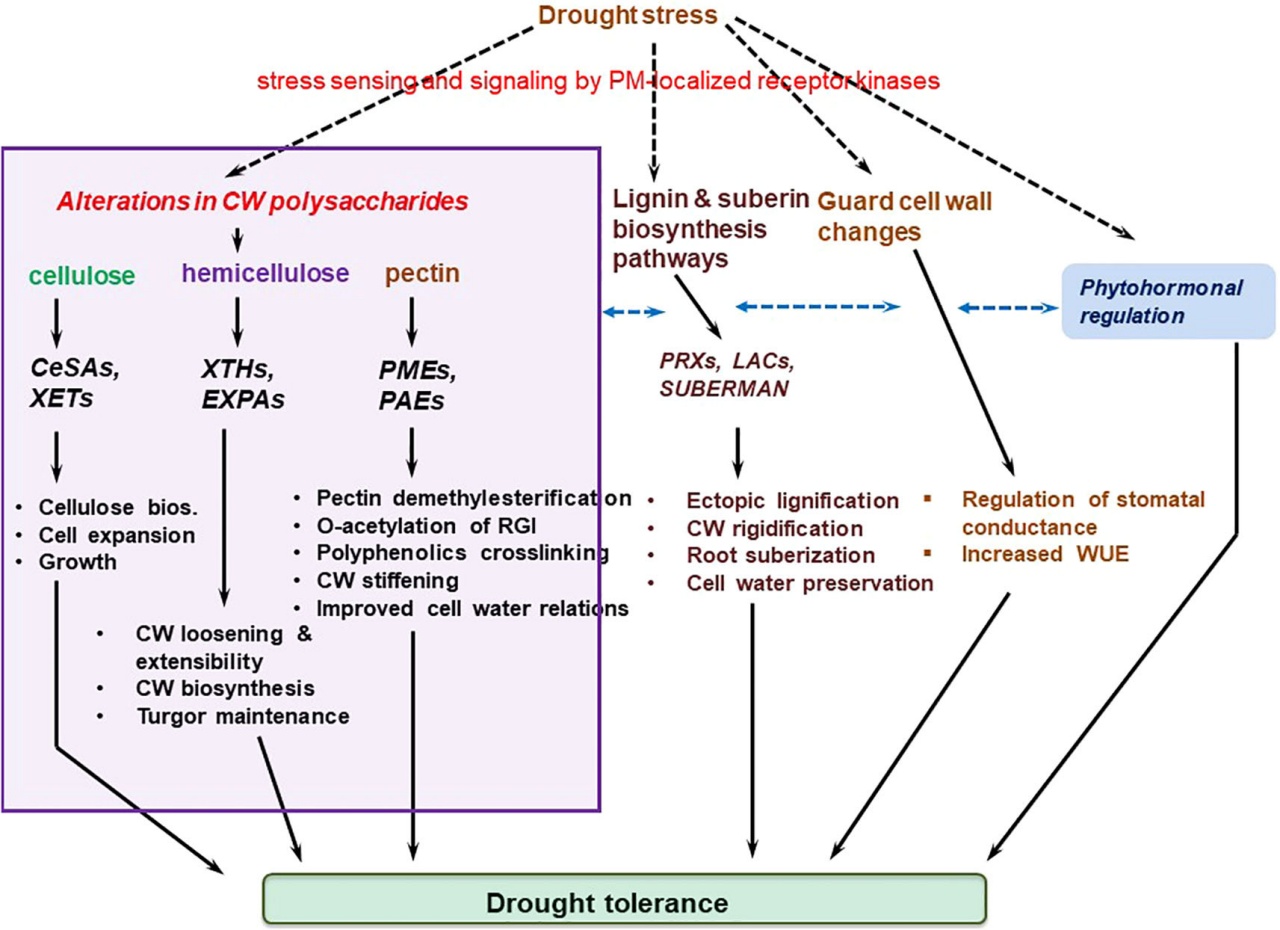
Summary of cell wall remodeling mechanisms orchestrating plant drought tolerance discussed in this review. Purple enclosure signifies cell wall integrity maintenance pathway, black dashed arrows represent stress sensing and signaling mediated by plasma membrane-localized receptor kinases/proteins, whereas blue dashed connectors represent crosstalk among the different signaling pathways. CeSA, cellulose synthase A; cellulose bios., cellulose biosynthesis; EXPAs, expansins; LACs, laccases; PAEs, pectin acetylesterases; PMEs, pectin methylesterases; PRXs, peroxidases; RGI, rhamnogalacturonan I; SUBERMAN, SUBERMAN transcription factor; WUE, water use efficiency; XETs, xyloglucan endotransglucosylases; XTHs, xyloglucan endotransglucosylases/hydrolases.

Cell wall interfaces the cell interior with its surrounding environment, regulating stress sensing, signaling and response; therefore, cell wall plasticity under drought stress crucially helps in regulating plant stress acclimation and adaptation. Here, we have synthesized how several cell wall remodeling mechanisms orchestrate drought tolerance in plants (**Figure 8**). Stress-induced demethylesterification of pectins, mediated by PMEs, permits calcium crosslinking of polyphenolics, which enhances cell wall rigidity and may help in intra-cell water preservation. Lignin accumulation-mediated cell wall thickening increases the plant tissue`s structural robustness, and regulates cell water movement across the cell membrane, which potentiates better plant tolerance to drought. Root suberization creates a hydrophobic and protective secondary cell wall layer that minimizes water loss and enhance drought tolerance. Similarly, PMEs and expansin activities that regulate guard cell wall plasticity are essential in controlling stomatal aperture in response to drought stress, and are excellent targets for genetic engineering of drought tolerance in crops. At the same time, highly plastic transcriptional regulation of secondary cell wall biosynthetic or stress-responsive genes, modulated by several TFs such as MYBs, NACs, WRKYs, etc., orchestrates plant drought tolerance. Meanwhile, phytohormones such as BRs, auxins, etc., crosstalk with ROS and cell wall integrity pathways to modulate stress response and transcriptional output of some cell-wall remodeling-associated genes. However, fine-tuning plant cell wall properties to engineer stress tolerance requires a deep understanding of these cell wall biosynthesis and regulation mechanisms, and then deploy genome engineering techniques to tailor the desired modifications. Harnessing modern high-resolution plant cell wall imaging techniques, single cell omics, genome editing and synthetic biology approaches could help us achieve this feat.
